# Biomaterials as Regulators of Neuron–Glia Crosstalk: From Material Properties to Functional Outcomes

**DOI:** 10.3390/cells15212019

**Published:** 2026-11-05

**Authors:** Valeriia S. Shtol, Sophia P. Konovalova, Kirill A. Arsentiev, Yulia I. Taranukha, Pavel E. Musienko

**Affiliations:** 1 Scientific Center for Genetics and Life Sciences, Sirius University of Science and Technology, 1, Olympic Ave., 354340 Sochi, Russia; konovalova.sp@talantiuspeh.ru (S.P.K.);; 2 Institute of Translational Biomedicine, St. Petersburg State University, 199034 St. Petersburg, Russia; 3 Moscow Center for Advanced Studies, 20, Kulakova Str., 123592 Moscow, Russia; 4 Pavlov Institute of Physiology, Russian Academy of Sciences, 199034 St. Petersburg, Russia

**Keywords:** biomaterials, neuron–glia interactions, neuron–glia crosstalk, material–tissue interface, neural tissue engineering, neural regeneration, neural interfaces, neuroinflammation, astrocytes, microglia

## Abstract

**Highlights:**

**What are the main findings?**

- The material–tissue interface integrates physicochemical material properties into neuronal, astrocytic, and microglial responses, but direct material effects and glia-mediated neuronal effects remain insufficiently distinguished in current studies.
- Mechanical, architectural, chemical, electrical, and degradation-related cues engage interconnected adhesion, mechanosensitive, and transcriptional signaling pathways, including FAK/Src, Piezo1/Ca^2+^, RhoA/ROCK, and YAP/TAZ signaling.

**What are the implications of the main findings?**

- Biomaterial design should be application-dependent. Regenerative systems require permissive and remodelable interfaces, whereas chronic neural interfaces require stable functional coupling while limiting adverse tissue responses.
- Progress toward predictive biomaterials requires multicellular models that resolve neuron–astrocyte–microglia–ECM interactions across physiologically relevant timescales rather than relying primarily on isolated cellular endpoints.

**Abstract:**

Biomaterials for neural tissue engineering, cell delivery, and chronic neural interfaces are conventionally characterized through physicochemical descriptors such as stiffness, topography, surface chemistry, and degradation rate. However, their biological effects are mediated by the material–tissue interface, which evolves through protein adsorption, extracellular matrix ligand presentation, and progressive cellular remodeling and determines how these design parameters are translated into neuronal and glial responses. This review synthesizes current evidence on how neurons, astrocytes, and microglia sense the material–tissue interface, how integrin-dependent adhesion complexes, mechanosensitive Piezo1 channels, and downstream FAK/Src, RhoA/ROCK, and YAP/TAZ pathways transduce interfacial cues into intracellular signaling, and how key design variables (mechanical properties, architecture, surface chemistry, electrical conductivity, and degradation) can be tuned to direct these processes in regenerative scaffolds and chronic neural interfaces. Particular attention is given to studies distinguishing direct neuronal effects from glia-mediated modulation, since concurrent changes in neuronal and glial markers are often reported without establishing causal neuron–glia interaction. We further discuss methodological limitations that constrain current interpretations of biomaterial-induced neuron–glia crosstalk, including discrepancies between 2D, 3D, and in vivo models and insufficient durations of chronic observation. We argue that functional biomaterial design for neural applications should move from optimizing isolated cellular readouts toward multicellular experimental frameworks capable of resolving neuron–glia–ECM interactions over physiologically relevant timescales.

## 1. Introduction

Disorders affecting the nervous system impose a substantial global health and economic burden. In 2021, these conditions affected an estimated 3.4 billion people and accounted for 443 million disability-adjusted life years worldwide, with stroke contributing the largest share [1]. Traumatic brain injury affects more than 50 million people annually, with an estimated global economic burden of US$400 billion per year [2]. Spinal cord injury affected approximately 20.6 million people worldwide in 2019 [3], with reported lifetime costs ranging from US$0.7 to 2.5 million per individual [4]. Biomaterial-based approaches to neural tissue repair, cell delivery, and implantable neural interfaces are being developed to address different aspects of these conditions [5,6]. Although economic evidence for emerging regenerative approaches remains limited, clinically established implantable technologies provide relevant examples. A systematic review of 14 economic evaluations of spinal cord stimulation reported cost-effective or cost-saving outcomes in 10 studies [7]. While these findings cannot be extrapolated to regenerative biomaterials, they illustrate the economic considerations associated with the clinical implementation of neural technologies. In both regenerative and implantable applications, the physicochemical and mechanical properties of biomaterials influence their interactions with neural tissue, including neuronal and glial responses that contribute to functional outcomes.

### 1.1. The Material–Tissue Interface as a Determinant of the Biological Response

Biomaterials for neural tissue repair, cell delivery, neuroprosthetics, and chronic neuromodulation have traditionally been described in terms of their physicochemical properties, including chemical composition, stiffness, topography, porosity, degradation rate, electrical conductivity, and other parameters [8]. However, the biological response to a material cannot be predicted solely from these properties. Upon contact with a biological environment, the material surface rapidly changes through protein adsorption and subsequent interactions with extracellular matrix (ECM) components. The composition, conformation, and accessibility of adsorbed molecules depend on the chemical and physical properties of the material and determine which receptor-binding sites are available for cellular recognition [9,10,11,12]. Therefore, the biological effects of a material are more appropriately considered in the context of the resulting material–tissue interface, whose properties may differ substantially from those of the original material surface [13,14].

This perspective is particularly important for neural tissue because biomaterials interact with multiple cell populations. Neurons, astrocytes, and microglia differ in how they sense their microenvironment and respond to changes within it; however, within tissue, these responses are not independent. Microglia and astrocytes contribute to the regulation of the inflammatory, trophic, and extracellular microenvironment of neural tissue, whereas neuronal survival and neurite growth depend, among other factors, on trophic support, interactions with the ECM, and electrical activity [15,16,17]. Intercellular communication can propagate and amplify changes in cellular states across different cell populations. Current studies of neuron–glia crosstalk, including microglia–neuron and microglia–astrocyte signaling axes, demonstrate that such intercellular regulation relies on specific ligand–receptor mechanisms [18,19]. For example, activated microglia have been shown to induce a distinct reactive astrocyte state through IL-1α, TNF, and C1q [20]. More recent evidence has also revealed direct mechanisms of bidirectional communication between microglia and neurons. A recently identified mechanism involves microglial secretion of β-hexosaminidase, which is taken up by neurons and contributes to the degradation of ganglioside GM2. When this process is disrupted, GM2 accumulates in neurons and, in turn, activates microglia via MGL2, thereby establishing a bidirectional intercellular loop [21]. Although these studies do not directly address biomaterials, they demonstrate a property of neural tissue that is fundamentally important for biomaterial evaluation: a change in the state of one cell population can secondarily alter the state of others.

This level of biological organization remains insufficiently represented in biomaterials research. In many studies, neuronal and glial responses are characterized using separate endpoint measures, such as cell viability, neurite growth, GFAP or Iba1 expression, and concentrations of inflammatory mediators. These measures can characterize the states of individual cell populations but do not establish causal relationships between their changes. Therefore, concurrent changes in neuronal and glial markers following contact with a material do not, by themselves, provide evidence of regulated neuron–glia interactions. A methodological gap thus remains between the well-established biology of neuron–glia crosstalk and its direct investigation in the context of biomaterials.

In this review, the material–tissue interface is considered the link between engineered material properties and the neural tissue response. We sequentially examine the formation of the material–tissue interface, its recognition by cells, the intracellular mechanisms that transduce interfacial signals, and the effects of material properties on neuronal, glial, and tissue responses. In doing so, we distinguish between effects directly demonstrated in individual cell types and evidence that provides insight into their intercellular interactions. This framework is subsequently used to compare two functionally distinct applications: neural tissue regeneration and long-term interactions with chronic neural interfaces.

Previous reviews have addressed biomaterial interactions with neural tissue, including neurogenic signaling at material interfaces [22], reactive astrogliosis [15], glial responses to implanted electrodes [23], and soft neural interfaces [6]. However, the extent to which material-induced neuronal and glial responses reflect changes in intercellular communication remains insufficiently resolved. This review critically examines the evidence linking biomaterial properties to neuron–glia interactions, distinguishing parallel cellular responses from experimentally supported intercellular mechanisms. Particular attention is given to how material-dependent changes in glial function may influence neuronal outcomes and to the implications of these interactions for regenerative scaffolds and chronic neural interfaces. By integrating mechanistic evidence with application-specific requirements, we identify key considerations for biomaterial design and experimental evaluation.

### 1.2. Biomaterials for Regeneration and Implantation

**Figure 1:**
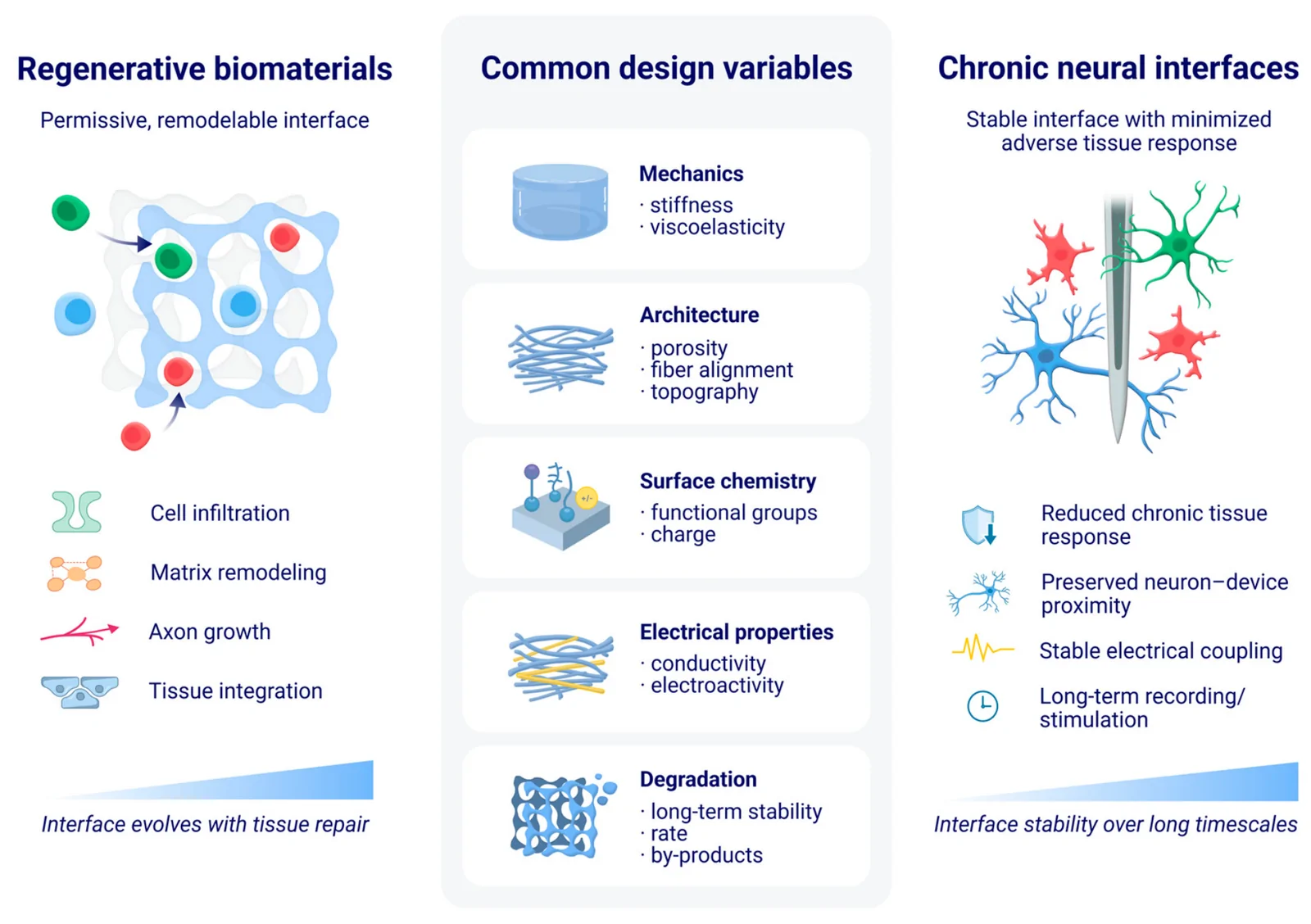
Application-dependent design of neural biomaterials: shared design variables and divergent interface requirements.

Biomaterials that interact with neural tissue are used in systems designed to serve fundamentally different functional purposes [22] (Figure 1).

In regenerative approaches, the material must provide an environment conducive to the repair of damaged tissue. Depending on the specific strategy, this requires not only biocompatibility and the absence of toxicity, but also support for cell adhesion, survival, and differentiation, neurite growth, directed cell migration, ECM organization, and modulation of the glial response in a manner favorable to the restoration of neuronal connections. Contemporary regenerative systems combine these functions with the delivery of cells, neurotrophic factors, and other bioactive components, while scaffold properties are used to guide the process of tissue repair [24,25].

Chronic neural interfaces serve a different functional purpose. An implant must maintain long-term mechanical and functional coupling with neural tissue, enabling stable recording or stimulation while minimizing damage to surrounding structures. Mechanical mismatch between the implant and soft neural tissue, micromotion, and chronic inflammation can disrupt this coupling and reduce the long-term performance of the device. The potential to mitigate these effects through mechanically compliant materials was demonstrated with the soft multimodal e-dura implant [26]. Contemporary bioelectronic systems extend this approach through the use of soft and stretchable materials, bioactive coatings, and hybrid architectures designed to promote more stable interactions between the device and surrounding tissue [6].

Thus, materials for regeneration and cell therapy and those used in chronic neural interfaces impose different requirements on the material–tissue interface. In the former context, the material should support cell invasion, structural organization, and remodeling of damaged tissue and, in some systems, gradually give way to the newly formed matrix. In the latter, the artificial interface must persist over extended periods and maintain functional interactions with neural tissue while minimizing processes that could compromise device performance. These requirements, however, cannot be reduced to a simple opposition between “integration” and “isolation.” Chronic neural interfaces also require close functional coupling with neurons, whereas excessive or nonselective cellular responses can lead to unfavorable tissue remodeling and impaired device performance.

The regulation of adhesion clearly illustrates this context dependence. In regenerative scaffolds, controlled cell attachment is generally required for cellular infiltration of the material, migration, and subsequent tissue organization. In chronic implants, by contrast, nonspecific protein adsorption and undesirable cellular overgrowth can contribute to an unfavorable tissue response and reduce the stability of the functional interface. The design objective is therefore neither to maximize nor to completely suppress adhesion, but rather to control its selectivity, spatial organization, and dynamics according to the intended function. In this review, regenerative and chronic systems are considered not as opposite ends of a universal adhesion axis, but as two distinct contexts in which the same fundamental processes governing the formation and cellular recognition of the material–tissue interface must lead to different functional outcomes.

## 2. Material–Tissue Interface: From Protein Adsorption to Cellular Recognition

### 2.1. Protein Adsorption and Formation of the Interfacial Protein Layer

One of the earliest events following contact between a biomaterial and a biological environment is protein adsorption onto its surface. Cells therefore interact not only with the underlying material but also with a dynamically evolving layer of adsorbed biomolecules. In nanoparticle research, this layer is commonly referred to as the protein or biomolecular corona [12,27]. For consistency, we use the term interfacial protein layer when discussing the implants, scaffolds, and coatings that are the primary focus of this review. In either context, the properties of the adsorbed layer depend not only on protein abundance but also on its molecular composition and the orientation and conformation of individual proteins.

The composition of the interfacial protein layer changes over time. In multicomponent biological environments, proteins compete for available surface sites, and previously adsorbed molecules can be displaced by others [28]. This process, known as the Vroman effect, was originally described for blood plasma proteins and is relevant to materials exposed to plasma, cerebrospinal fluid, or injured tissue, where albumin, fibrinogen, immunoglobulins, and complement components may coexist [29]. Experimental studies have shown that the composition of the interfacial layer depends on protein concentration, relative surface affinity, molecular characteristics, and adsorbent properties [30]. More recent analyses indicate that competitive protein exchange involves mechanisms more complex than the sequential replacement of lower-affinity proteins by those with higher surface affinity [31].

Protein adsorption is strongly influenced by the physicochemical properties of the surface. Hydrophobicity, surface energy, charge, and functional group chemistry affect both the amount of protein adsorbed and its state following adsorption. For example, systematic studies of surfaces with different wettabilities have demonstrated a pronounced dependence of protein adsorption on surface energy [32]. However, the amount of adsorbed protein alone is insufficient to predict the cellular response. Interactions with the surface can alter protein conformation, thereby exposing or, conversely, masking sites available for recognition by cellular receptors.

The functional significance of protein conformation has been demonstrated in studies of fibronectin adsorption onto model surfaces bearing different terminal functional groups. Surface chemistry altered the accessibility of the central cell-binding domain of adsorbed fibronectin, resulting in differences in integrin binding and cell adhesion [33]. These findings illustrate why comparable amounts of adsorbed protein may produce different cellular responses: receptor recognition depends on the accessibility of specific binding sites within the interfacial protein layer. Surface physicochemical properties can thereby influence cellular signaling through changes in the molecular presentation of adsorbed proteins.

The dynamics of the material–tissue interface are particularly important in the central nervous system (CNS), where the local microenvironment depends on the state of the tissue. The foreign body response to implanted hydrogels is influenced by their physicochemical properties and recapitulates multicellular responses characteristic of CNS injury. Both the nature of the response and the functional properties of the hydrogels differed when they were introduced into the intact brain versus the site of ischemic injury [34]. These findings emphasize that biomaterial interactions with neural tissue depend not only on the intrinsic properties of the material but also on the state of the surrounding tissue. Surface characteristics established under standard in vitro conditions therefore cannot be directly equated with those encountered following in vivo implantation.

The next level of interaction is determined by how the molecular motifs presented at this interface are recognized by cellular receptors and translated into adhesive signaling.

### 2.2. Cellular Recognition of ECM Ligands and Formation of Adhesion Complexes

**Figure 2:**
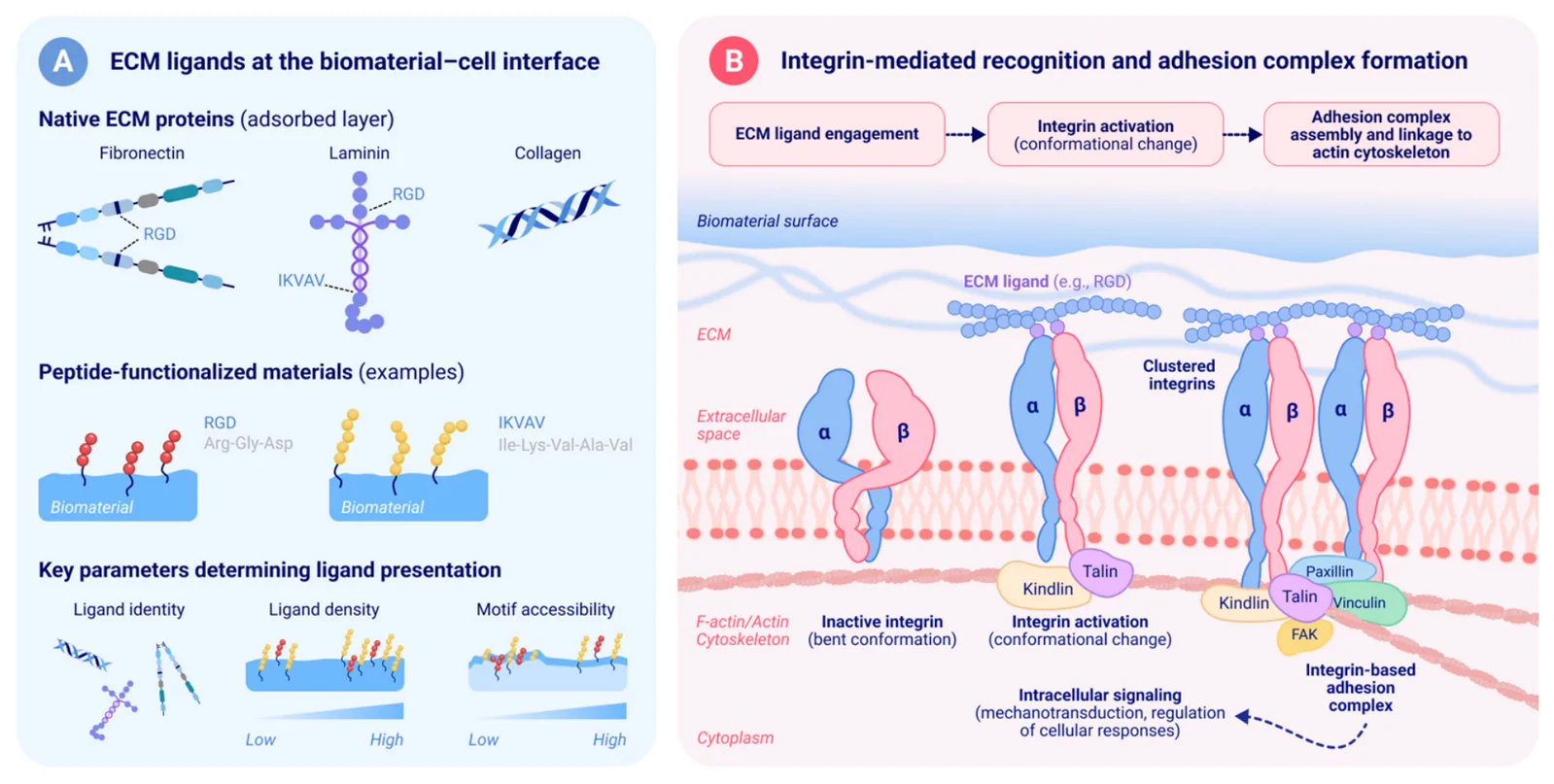
ECM ligand presentation and integrin-mediated adhesion complex formation at the biomaterial–cell interface. (**A**) The presentation of ECM ligands is determined by the composition and organization of adsorbed proteins, including fibronectin, laminin, and collagen, as well as by the incorporation of bioactive peptide motifs, such as RGD and IKVAV, into biomaterial surfaces. Ligand identity, surface density, and accessibility of recognition sites influence subsequent cellular interactions. (**B**) Integrins recognize specific ECM ligands and undergo conformational changes associated with receptor activation and clustering. Recruitment of intracellular adaptor proteins, including talin, kindlin, paxillin, and vinculin, promotes the assembly of adhesion complexes and their linkage to the actin cytoskeleton. These interactions contribute to mechanotransduction and downstream signaling pathways involved in the regulation of cellular responses. The schematic illustrates representative molecular interactions and does not imply that all ECM-derived peptide motifs engage integrins through identical mechanisms.

Integrins are key receptors involved in the recognition of ECM components. These heterodimeric transmembrane proteins mechanically and functionally link the ECM to the actin cytoskeleton [35]. Different integrin heterodimers exhibit distinct ligand specificities and interact with fibronectin, laminins, collagens, and other ECM components. Cellular recognition of the interface therefore depends not only on the composition of the interfacial protein layer, but also on the accessibility of specific receptor-binding sites and the repertoire of integrins expressed by the cell (Figure 2).

In neurons, integrin-dependent recognition of the ECM is directly associated with neurite growth. In adult sensory neurons, culture on laminin enhanced neurite outgrowth, whereas β1-integrin blockade attenuated this effect. The addition of nerve growth factor (NGF) further enhanced integrin-dependent growth, indicating functional interactions between adhesive and neurotrophic signals [36]. These findings demonstrate that neuronal interactions with the ECM are not limited to mechanical attachment but also contribute to the regulation of cellular responses to other signals within the microenvironment.

The ability of cells to recognize specific ECM motifs is exploited in the functionalization of biomaterials with short peptide sequences that mimic biologically active regions of the native ECM. Rather than incorporating full-length ECM proteins, materials can be functionalized with short peptide sequences representing individual receptor-active sites. Among the most widely used are RGD-containing sequences and laminin-derived peptides, including IKVAV. This approach enables the biological activity of a synthetic matrix to be modified without reproducing the full molecular composition of the native ECM. The laminin-derived IKVAV motif is among the most extensively studied in neural tissue engineering. An IKVAV-functionalized poly(lactide ethylene oxide fumarate) (PLEOF) hydrogel supported the attachment, growth, proliferation, and differentiation of neural stem cells (NSCs). The resulting NSC spheroids could also be encapsulated within the three-dimensional hydrogel, followed by cell attachment and proliferation [37].

The biological effect, however, is determined not only by the presence of a particular motif but also by its density and mode of presentation. This has been clearly demonstrated using IKVAV-functionalized PEG hydrogels. Immobilization of a short IKVAV peptide increased the attachment and proliferation of human neural stem/progenitor cells (hNSCs) and promoted their migration into the hydrogel. Maximum migration was observed at a peptide density of 10 μM, whereas higher concentrations had an inhibitory effect and resulted in shorter migration distances. Differences in cellular responses among the IKVAV-containing sequences examined were attributed to differences in motif accessibility to cellular receptors. Importantly, the stiffness of the peptide-modified hydrogels remained similar, at approximately 600 Pa, allowing the observed differences to be attributed primarily to changes in ligand functionalization rather than to the mechanical properties of the matrix. These findings demonstrate that increasing the amount of an adhesive ligand does not necessarily result in a proportional enhancement of the cellular response [38]. For biomaterials used in neuroscience, the effectiveness of such functionalization therefore cannot be evaluated solely in terms of the overall ability of the material to support cell adhesion. Ligand specificity, density, and motif accessibility determine which receptor interactions can occur. Moreover, a single material interacts with multiple cell populations that differ in both the repertoire and functional roles of their adhesion receptors. The same molecular interface may therefore elicit different responses in neurons and glial cells.

Binding of ECM ligands is accompanied by integrin activation and the organization of integrin-containing adhesion complexes. Talin and kindlin play key roles during the early stages of this process by interacting with the cytoplasmic domains of integrins and contributing to their activation and the assembly of nascent focal adhesions. The emerging network of protein interactions involving vinculin, paxillin, and other components links integrins to the actin cytoskeleton and supports the subsequent organization of focal adhesions [39]. These complexes serve not only a structural function but also provide a platform for transducing signals from the material–tissue interface into intracellular responses. The mechanisms underlying subsequent signal transduction and mechanotransduction are discussed in Section 4.

### 2.3. Dynamic Remodeling of the Material–Tissue Interface

Following cellular recognition, the material–tissue interface continues to evolve. This is particularly evident in three-dimensional and biodegradable matrices, whose structure can be remodeled directly by cells. In proteolytically remodelable hydrogels, cells locally degrade the polymer network, altering the space available for cell spreading and the conditions for intercellular interactions. Material degradation therefore represents not only a determinant of material lifetime but also a parameter governing the ability of cells to remodel their immediate microenvironment. In the context of neural cells, the role of such remodeling was demonstrated in a series of experiments using neural progenitor cells (NPCs) encapsulated within three-dimensional elastin-like protein hydrogels [40,41].

In the first study, independent variation in hydrogel stiffness and proteolytic degradability showed that, across an initial stiffness range of approximately 0.5–50 kPa, maintenance of NPC stemness did not correlate with initial hydrogel stiffness but required the ability of the cells to remodel the matrix [40]. In poorly degradable hydrogels, NPCs retained a rounded, isolated morphology, whereas in highly degradable scaffolds, the cells remodeled the surrounding network and established intercellular contacts. Blocking degradation through protease inhibition or ADAM9 knockdown reduced proliferation. Mechanistically, this effect was associated with the formation of cadherin-dependent cell–cell contacts and β-catenin signaling rather than with differences in adhesive ligand presentation or initial hydrogel stiffness [40]. Thus, in a three-dimensional neural system, the ability of cells to physically remodel the material can determine the accessibility of subsequent intercellular signaling.

A subsequent study using the same experimental platform showed that remodeling was required not only for the maintenance of NPCs but also for their subsequent differentiation. Even in highly degradable hydrogels, induction of differentiation after only one day of remodeling resulted in a substantially weaker response than induction after seven days of prior remodeling. Following sufficient matrix remodeling, NPCs differentiated into both astrocytes and neurons expressing βIII-tubulin, MAP2, doublecortin, and neurofilament; the resulting neurons exhibited calcium transients in response to stimulation with GABA and glutamate. This effect was linked to cadherin-dependent regulation of YAP expression. Thus, for a 3D matrix, not only its initial composition but also the ability of cells to alter the local material structure over time is important [41].

The importance of dynamic remodeling is also supported by in vivo studies, where host tissue cells contribute to the restructuring of the interface. The thermosensitive imidazole-poly(organophosphazene) hydrogel I-5 was investigated in a rat model of contusive spinal cord injury [42]. Four weeks after hydrogel administration, the volume of cystic cavities was reduced from 1.00 to 0.14 mm^3^, and the lesion area was filled with a fibronectin-rich ECM. Macrophage involvement was critical to this process. Macrophages expressed MMP-9 within the remodeling fibrous tissue, whereas a control poly(organophosphazene) hydrogel lacking the imidazole group required for interaction with macrophages through histamine receptors induced ECM bridge formation to a substantially lesser extent. Thus, in this model, the material did more than simply fill the lesion cavity: its interactions with inflammatory cells altered the organization of the extracellular microenvironment at the implantation site.

A different form of interface dynamics has been demonstrated for ECM-derived materials in the injured brain. In a post-stroke cavity model, hydrogels derived from decellularized urinary bladder matrix exhibited concentration-dependent differences in biodegradation and the associated tissue response [43]. By 90 days, the volumes of the 3 and 4 mg/mL hydrogels had decreased by approximately 95%, whereas the more concentrated and stiffer 8 mg/mL hydrogel decreased in volume by approximately 32%. Hydrogels that underwent extensive degradation were characterized by cellular infiltration, the presence of macrophages and endothelial cells, neovascularization, and replacement of the implantation site with newly formed tissue. In contrast, the stiffer and more slowly degrading formulation did not support a comparable degree of tissue restoration [43]. These findings demonstrate that the properties of the material–tissue interface in vivo can diverge over time even among materials of the same origin because of differences in their degradation and subsequent cellular colonization.

Taken together, these findings refine the linear framework presented in the preceding subsections. Protein adsorption and recognition of ECM ligands determine the early stages of cell–material interactions, but the resulting interface subsequently becomes a target of cellular remodeling itself. The nature of this feedback depends on the system. In synthetic proteolytically responsive hydrogels, NPCs directly modify the surrounding polymer network, affecting intercellular contacts and subsequent cell fate [40,41]. Following implantation into the injured CNS, this process is further shaped by inflammatory and vascular cells, proteolytic activity, material degradation, and ECM reorganization [42,43]. The material–tissue interface should therefore be regarded not as a fixed boundary with properties predetermined during fabrication, but as a dynamic system whose state is determined by both the material and cellular activity.

## 3. Cell-Specific Sensing of the Material

### 3.1. Cell-Specific Sensing and Biomaterial-Mediated Neuron–Glia Interactions

**Figure 3:**
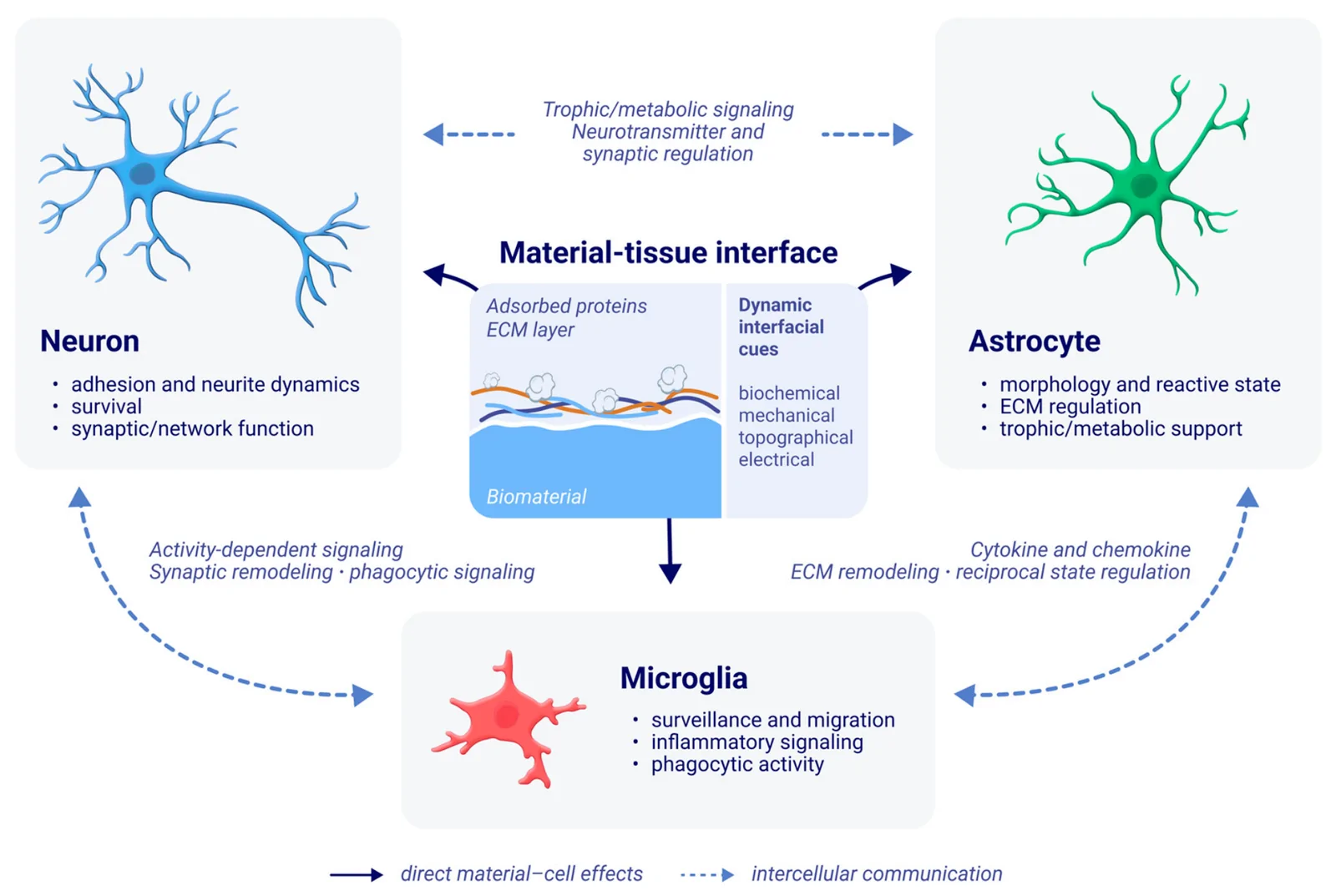
Cell-specific responses of neurons, astrocytes, and microglia to the material–tissue interface and potential routes of intercellular signaling among these cell populations.

The material–tissue interface simultaneously interacts with cell populations that differ in their receptor repertoire, cytoskeletal organization, mechanosensitivity, and functional roles within neural tissue (Figure 3). In neurons, substrate properties are directly associated with growth cone formation, neurite extension and branching, and the formation of neuronal networks [44]. In astrocytes, these properties can alter morphology, reactivity, and functions involved in maintaining the neuronal microenvironment [45,46,47]. Microglia, in turn, can translate the mechanical and adhesive properties of their microenvironment into changes in morphology, migration, and immune responses [48,49,50].

Material nanotopography can differentially affect neuronal and astrocytic surface coverage in mixed cultures. In primary neuron–glia co-cultures derived from the perinatal rat neocortex, nanoporous gold (np-Au) reduced the surface area covered by GFAP-positive astrocytes by 48–57% relative to planar gold, whereas neuronal coverage remained at approximately 36% on both surfaces [51]. Experiments using Al_2_O_3_-coated substrates and varying residual silver concentrations helped distinguish the effects of nanotopography from those of surface chemistry and potential silver-related toxicity. Astrocyte density and proliferation were not reduced, suggesting that the decrease in astrocytic surface coverage reflected changes in cell spreading rather than cell loss. These findings demonstrate a differential response of neuronal and astrocytic components to the material surface but do not establish whether neuron–glia interactions were affected. In a subsequent study, the same group reported that np-Au-conditioned medium did not reproduce the reduction in astrocyte coverage, although adhesion of astrocytes to np-Au itself was reduced [52]. These observations are consistent with a direct effect of nanotopography on astrocyte adhesion, although they do not exclude other mechanisms contributing to the cellular response.

At a more complex level of material organization, embryonic rat cortical neurons, together with glia that developed in culture without antimitotic treatment, were grown on multielectrode arrays coated with brain extracellular matrix derived from decellularized tissue (bECM), MaxGel, a non-tissue-specific ECM, or poly-D-lysine. Both ECM coatings accelerated the development of synchronized network activity relative to poly-D-lysine, although their effects differed. At 23 days in vitro (DIV), bECM-coated arrays showed a greater spatial extent of network activity, with at least half of the electrodes active compared with approximately one quarter on the other coatings. MaxGel increased the network burst rate and was associated with a nonsignificant trend toward higher synaptophysin expression. Differences in the proportion of active electrodes were no longer detectable by approximately 30 DIV. No differences were detected in the neuron-to-glia ratio, glial proliferation, or the assessed GFAP and nestin expression [53]. Thus, differences in network activity were observed without corresponding changes in the measured cellular composition or glial markers. However, the experimental design did not establish whether these differences resulted from direct effects of the ECM coatings on neurons, changes in glial function, or both.

Distinguishing between direct neuronal responses and glia-mediated effects becomes particularly important when a material is considered a regulator of neuron–glia crosstalk. Evidence for an astrocyte-mediated contribution to neuronal responses comes from experiments in which primary rat astrocytes were exposed to graphene oxide (GO) flakes, internalized through the endolysosomal pathway, before subsequent co-culture with primary neurons [54]. Prolonged GO exposure altered astrocyte morphology and was accompanied by increased inward-rectifying K^+^ channel activity and enhanced Na^+^-dependent glutamate uptake. These changes were followed by increased neuronal excitability and GABAergic synapse density in co-culture. Unlike studies reporting parallel neuronal and glial responses to a material, this experimental design involved pretreatment of astrocytes before their co-culture with neurons. However, GO was applied as dispersed flakes rather than as a substrate, and the study did not identify the mediators responsible for the neuronal changes or establish whether altered K^+^ channel activity or glutamate uptake was necessary for these effects.

The effects of matrix properties on astrocyte-mediated neuronal responses have also been investigated using conditioned-medium experiments. Human iPSC-derived astrocyte progenitors were cultured within hyaluronic acid scaffolds differing in stiffness and functionalized with collagen IV and fibronectin (Coll-IV/Fn). Soft Coll-IV/Fn-functionalized scaffolds (<1.5 kPa) supported astrocyte progenitor growth and enhanced their neurotrophic activity. Conditioned medium from these scaffolds increased average neurite length in NSC-34 motor neuron-like cells by 3.4-fold relative to unconditioned controls, with significantly greater neurite extension than that observed with conditioned medium from stiffer or non-functionalized scaffolds. Similar effects on neurite extension were observed in primary mouse spinal cord neurons [55]. The study included cell-free scaffold-conditioned medium controls, supporting a contribution from factors released by the scaffold-associated astrocyte progenitors rather than scaffold components alone. However, the molecular mediators responsible for the enhanced neurite growth were not established through selective inhibition or rescue experiments.

Material-induced changes in astrocytes can affect neurons not only through homeostatic functions but also through alterations in glial morphology and phenotype. Astrocytes from the postnatal mouse cortex were cultured on poly(methyl methacrylate) (PMMA) substrates patterned with microgrooves 2 or 10 μm in width. On the 2-μm pattern, astrocytes acquired an elongated morphology, showed increased expression of nestin and Pax6, proliferated, and exhibited features resembling radial glial cells. Although astrocytes also aligned on wider grooves, these phenotypic changes were observed on the 2-μm pattern, whose dimensions were comparable to those of radial glial processes. When embryonic cortical neurons or cortical explants were subsequently seeded onto the patterned glial layer, oriented axonal outgrowth and neuronal migration were observed, including in time-lapse imaging experiments [56]. These findings demonstrate that material topography can influence astrocyte morphology and phenotype in a culture system that supports directed neuronal growth and migration. However, the experiments do not establish whether the neuronal responses depended specifically on the altered glial phenotype, the organization of the glial layer, direct contact guidance by the patterned substrate, or a combination of these factors.

Surface chemistry can also influence astrocytic responses to fibrous scaffolds. Polycaprolactone (PCL) nanofibers were functionalized with poly(L-lysine)–lactobionic acid (PLL–LBA), introducing galactose moieties onto the material surface. In vitro, galactose functionalization was associated with lower astrocytic levels of GFAP, AHNAK, F-actin, and Gal-1 during long-term culture compared with unmodified PCL nanofibers and nanofibers functionalized with heparin and PLL without galactose moieties [57]. These changes were not accompanied by a reduction in astrocytic L-glutamate transporter activity. Following implantation into the mouse striatum through a guide needle, producing a stab-type injury, galactose-functionalized scaffolds were associated with greater numbers of NeuN-positive neurons both near and farther from the implant at day 7, alongside greater numbers of GFAP-positive astrocytes at the scaffold surface. These differences were no longer apparent at day 21. In vivo, however, the galactose-functionalized scaffolds were compared only with unmodified PCL, and three animals per group were analyzed at each time point. Thus, this study demonstrates that chemical surface modification can simultaneously alter astrocytic and neuronal responses to an implant, but does not establish a causal relationship between them.

The contribution of microglia to neuronal responses within biomaterial-based systems has also been examined through selective cell depletion. Three-dimensional engineered neural tissues were generated from primary mixed cortical cells of embryonic day 16–18 rats using silk fibroin/collagen composite scaffolds. Microglia were depleted from the initial cell population by magnetic separation using CD11b/c microbeads. Compared with constructs containing microglia, microglia-depleted constructs showed lower MAP2 expression at day 7 and synaptophysin expression at day 14, along with reduced levels of other neuronal and synaptic markers [58]. These findings support a contribution of microglia to the expression of markers associated with neuronal maturation and synaptic organization in this engineered tissue model. However, the scaffold properties were not varied, and the study therefore does not establish whether specific material characteristics regulate microglia–neuron interactions. Furthermore, neuronal function was not assessed electrophysiologically, and the mechanisms underlying the observed effects of microglial depletion were not identified.

The relationship between material architecture, microglial state, and the subsequent response of neural cells was examined by comparing two-dimensional graphene films with three-dimensional graphene foams in the BV2 microglial cell line [59]. In the absence of an inflammatory stimulus, neither graphene substrate induced a pronounced pro-inflammatory microglial response compared with tissue culture polystyrene. Following lipopolysaccharide (LPS) stimulation, the three-dimensional graphene foam, unlike 2D graphene, attenuated the characteristic morphological transformation of microglia and reduced the release of TNF-α and IL-1β and the expression of iNOS. Conditioned medium collected after culturing microglia on the different substrates was subsequently applied for 24 h to neural stem cells from the postnatal mouse hippocampus; medium from microglia cultured on 3D graphene foam increased their viability, and this difference was more pronounced after LPS stimulation [59]. The conditioned-medium experiments provide evidence that changes in the microglial response to graphene substrates are associated with altered neural stem cell viability. Although the study identified differences in TNF-α and IL-1β release and iNOS expression, it did not establish whether these changes were responsible for the observed effects on neural stem cells. Furthermore, the use of an immortalized microglial cell line and the assessment of neural stem cell viability without additional functional readouts limit the conclusions that can be drawn about microglia-mediated effects in primary neural tissue.

These studies differ in the strength of the evidence they provide for biomaterial-mediated neuron–glia interactions. Some report concurrent changes in neuronal and glial readouts without establishing a causal relationship between them. Others provide stronger evidence for glial contributions through sequential exposure and co-culture, conditioned-medium experiments, or selective glial depletion. For example, experiments involving GO-pretreated astrocytes [54], astrocyte progenitors cultured within Coll-IV/Fn-functionalized hyaluronic acid scaffolds [55], and conditioned medium from microglia cultured on graphene foam support the involvement of glial cells in subsequent neuronal or neural precursor responses, although the causal contributions of individual molecular mediators remain unresolved [59]. More direct mechanistic evidence was obtained in the patterned-laminin system, where antibody-blocking experiments demonstrated the contribution of astrocyte-associated fibronectin to neurite guidance [60]. In a different experimental context, astrocyte-specific Piezo1 deletion and ATP-mediated rescue established a role for astrocytic Piezo1-dependent ATP release in regulating adult neurogenesis [45]. This example illustrates the importance of testing specific intercellular signaling mechanisms, for which comparable causal evidence remains limited in the biomaterial-based studies discussed here.

Taken together, these studies demonstrate that cell-specific responses to the material–tissue interface can extend beyond the cell population in direct contact with the material. Evidence for glia-mediated contributions has been obtained in experimental systems using sequential exposure, co-culture, or conditioned-medium approaches [54,59]. Among these interactions, astrocytes occupy a particularly important position because of their direct involvement in the regulation of ionic and neurotransmitter homeostasis, trophic support, synaptic function, and ECM organization. The following section therefore examines which properties of the material–tissue interface determine astrocytic responses and how these responses should be interpreted in regenerative and chronic implant systems.

### 3.2. Astrocyte Sensing of the Material–Tissue Interface

Astrocytes are sensitive to several independently tunable properties of the material–tissue interface. Substrate stiffness can alter their adhesion, morphology, proliferation, GFAP expression, and functional properties, including glutamate uptake [46,47,61], whereas nanotopography regulates cell attachment, focal adhesion organization, and the extent of GFAP immunoreactivity [51,62]. The spatial organization of ECM ligands, in turn, can determine astrocyte orientation and the organization of the cell–matrix interface they form [60]. These responses do not necessarily change in parallel; therefore, astrocyte sensing of a material cannot be reduced to a single measure of reactivity.

The relationship between nanotopography and the astrocytic adhesion machinery was examined using anodic aluminum oxide surfaces with nanopore diameters of 20 and 90 nm [62]. Cortical astrocytes exhibited greater adhesion on surfaces with smaller pores and formed more vinculin-associated focal adhesions, predominantly distributed at the cell periphery, while simultaneously displaying lower GFAP immunoreactivity than cells on nonporous control surfaces or surfaces with 90-nm pores. Thus, enhanced adhesion is not necessarily accompanied by an increase in the GFAP-associated response.

Astrocyte sensitivity to the structural and physicochemical characteristics of a material is also evident in more complex fibrous systems. Primary cortical neuron–glia cultures on electrospun PLA/PCL scaffolds exhibited scaffold-dependent differences in the morphology of GFAP+ astrocytes, including cell shape and process branching characteristics [63]. On DCM/THF-derived scaffolds, astrocytes exhibited a less rounded morphology and more complex process organization, whereas cell rounding and process retraction were observed on several HFIP-derived matrices [63]. Unlike the nanoporous model surfaces described above, these fibrous matrices differed simultaneously in several physicochemical and structural characteristics; therefore, the contribution of any individual interface parameter cannot be isolated.

The spatial organization of adhesive cues can be transmitted through an astrocytic layer and thereby alter the neuronal microenvironment. Patterned laminin presentation aligned primary astrocytes, while dorsal root ganglion (DRG) neurons seeded onto the resulting astrocytic layer preferentially extended their neurites along the same axis [60]. Astrocyte-derived fibronectin also exhibited pronounced spatial anisotropy, and blocking fibronectin reduced the proportion of directionally extending neurites from more than 82% to 46%. Moreover, fibronectin alignment was maintained in astrocyte tissue constructs. Thus, the spatial organization of laminin on the material surface is transmitted through the aligned astrocytic layer and astrocyte-derived fibronectin, generating a secondary biological interface that guides neurite growth [60]. However, the signaling mechanisms underlying astrocyte alignment and fibronectin anisotropy were not identified.

The astrocytic response to substrate stiffness depends substantially on cell type and the surrounding cellular environment. The stiffness dependence of GFAP+ astroglial adhesion to polyacrylamide substrates differed between pure astroglial cultures and mixed neuron–astroglia cultures [46]. In mixed cultures, the greatest number of GFAP+ cells was observed at intermediate stiffness, whereas in astroglial monocultures, adhesion increased with increasing substrate stiffness. Moreover, on the soft 300-Pa substrate, where relatively few astrocytes were present, neurons were more susceptible to glutamate-induced toxicity, whereas this effect was attenuated on stiffer substrates. Thus, the response to the mechanical properties of the interface depends not only on stiffness itself but also on the cellular composition of the system, limiting the extent to which findings from astrocyte monocultures can be extrapolated to multicellular neural tissue.

Comparison of mechanosensitivity studies reveals another important limitation: the direction of the astrocytic response depends on the stiffness range examined and the experimental model. Primary rat astrocytes cultured on 8000-Pa polydimethylsiloxane (PDMS) substrates exhibited greater cell spreading and proliferation, GFAP expression, and ROS production, together with reduced glutamate uptake, compared with cells cultured on 200-Pa substrates [47]. In contrast, within the considerably narrower stiffness range of 300–1000 Pa, the highest GFAP immunoreactivity, proliferation, and process complexity were observed on the softest, 300-Pa substrate, whereas at 1 kPa astrocytes exhibited a morphology more closely resembling the basal state. The stiffness dependence of ECM-related markers followed a different pattern: the intermediate 800-Pa substrate increased the proportion of Aggrecan+, Brevican+, and Neurocan+ astrocytes [61]. These findings should not be regarded as directly contradictory, as the studies differed in the stiffness ranges examined, substrate materials, and experimental protocols. Taken together, they indicate that the available evidence does not support a universal relationship between substrate stiffness and astrocyte “reactivity”; rather, the structural, functional, and ECM-related components of the astrocytic response should be evaluated separately.

This distinction is particularly important when considering the consequences of material-induced astrocytic responses for neuronal function. Beyond changes in GFAP expression and morphology, astrocytes contribute to the maintenance of the neuronal microenvironment through Na^+^-dependent glutamate uptake, regulation of extracellular K^+^ concentrations, metabolic interactions with neurons, and the synthesis and remodeling of ECM components [64]. Such metabolic coupling may involve lactate transfer from astrocytes to neurons, although the relative contribution of this pathway varies with physiological conditions and remains under debate [65]. Astrocyte-associated ECM components, including chondroitin sulfate proteoglycans, can also influence axonal growth, but their effects depend on the molecular composition of the extracellular environment and the context of tissue injury [66].

Some of these functions have been examined in biomaterial-based experimental systems. For example, exposure to graphene oxide increased astrocytic inward-rectifying K^+^ channel activity and Na^+^-dependent glutamate uptake [54], whereas astrocytes cultured on stiffer PDMS substrates exhibited reduced glutamate uptake alongside increased GFAP expression and ROS production [47]. The stiffness-dependent differences in aggrecan, brevican, and neurocan expression described above [61] further indicate that ECM-related changes cannot be inferred from GFAP expression or cell morphology alone. However, changes in the expression of these proteoglycans do not necessarily establish alterations in ECM deposition, remodeling, or its functional effects on axonal growth. Likewise, changes in glutamate uptake or K^+^ channel activity do not, by themselves, establish whether the resulting astrocytic response is neuroprotective or neurotoxic. Astrocyte–neuron metabolic coupling, including lactate-mediated metabolic support, also remains insufficiently characterized in the biomaterial studies discussed here. A comprehensive assessment of astrocytic responses to biomaterials requires complementary molecular, morphological, and functional measurements, ideally accompanied by direct evaluation of neuronal outcomes. This interpretation is in line with current consensus recommendations on the characterization of reactive astrocytes, which emphasize that astrocyte states are heterogeneous and context-dependent and should not be defined by GFAP expression or morphological changes alone [67].

### 3.3. Microglial Sensing of the Material–Tissue Interface

Microglia exhibit a distinct mode of sensing the material–tissue interface that is closely linked to their immune functions. They rapidly respond to local neural tissue injury by extending their processes toward the lesion and migrating to the injured area [48,68]. Following implantation, microglia respond both to tissue injury caused by the implantation procedure and to the presence of the material itself and the resulting material–tissue interface [29,34]. Consequently, the microglial response observed in vivo cannot be unequivocally attributed to any single property of the implanted material.

Different measures of the microglial response to a material do not necessarily change in parallel. Surface chemistry influenced both the attachment of primary microglia and the secretion of IL-1β and TNF-α; however, the number of attached cells did not parallel the levels of cytokine secretion [49]. In the same study, CD11b+ cells remained directly associated with the surface of intracortical implants for up to 12 weeks after implantation. Thus, the presence of microglia at the material surface and their functional state represent distinct characteristics of the cellular response.

Microglia also sense the mechanical properties of their environment. Substrate stiffness altered cell spreading, actin cytoskeletal organization, and traction forces in primary microglia, while on stiffness-gradient substrates the cells preferentially migrated toward stiffer regions [50]. LPS activation, in turn, altered cellular mechanics and enhanced durotaxis, demonstrating that mechanical sensing depends on the functional state of the microglia themselves. A subsequent study showed that mechanical cues can affect not only microglial morphology and migration but also phagocytic activity and interactions with neurons [69]. Microglial mechanosensitivity is therefore an integral component of their functional response rather than an isolated reaction to the physical properties of the surrounding environment.

This multidimensionality limits the assessment of microglia–material interactions based on individual markers or a binary M1/M2 classification. Current nomenclature recognizes microglia as highly dynamic cells that exhibit multidimensional states, which should be characterized in terms of specific molecular and functional features [70]. In biomaterials research, this means that morphology, migration, phagocytosis, and mediator secretion should be evaluated as distinct and potentially discordant components of the microglial response. This approach is particularly important when interpreting data on materials capable of simultaneously affecting multiple microglial functions.

Functionally relevant aspects of microglial activity include the phagocytosis of cellular debris and apoptotic cells, the remodeling of synaptic connectivity, and the release of inflammatory and trophic mediators. In experimental models of mechanical injury, stretch applied to microglia increased their phagocytic activity and synaptic stripping in compartmentalized neuron–microglia co-cultures [69]. In three-dimensional engineered neural tissue based on silk fibroin/collagen scaffolds, microglial depletion reduced the expression of neuronal and synaptic maturation-associated markers, supporting a contribution of microglia to neural tissue development, although the underlying molecular mechanisms were not identified [58].

Microglial inflammatory signaling is also regulated by mechanosensitive pathways. Pharmacological activation of Piezo1 suppressed LPS-induced TNF-α and IL-6 production through a Ca^2+^-dependent mechanism involving inhibition of NF-κB signaling [71]. In a different experimental context, microglia-derived IL-1α, TNF, and C1q acted in combination to induce a neurotoxic reactive astrocyte state [20]. Although these studies did not directly address biomaterial-dependent neuron–glia signaling, they illustrate pathways through which microglial responses can shape the surrounding neural environment. Consequently, microglial density, morphology, or Iba1 immunoreactivity alone cannot establish whether a material-associated response is protective or detrimental to neural tissue.

These considerations are particularly important when reduced glial reactivity is interpreted as evidence of improved biocompatibility. Following CNS injury, certain astrocytic and microglial responses contribute to the formation of tissue boundaries and the containment of damage. Astrocyte-specific deletion of STAT3 disrupted astroglial scar formation and was associated with increased inflammatory cell infiltration and tissue damage after spinal cord injury [72]. Preventing astrocytic scar formation did not promote spontaneous axon regrowth and reduced the axonal regrowth elicited by specific growth-promoting interventions [73]. Similarly, microglial depletion following spinal cord injury disrupted glial scar formation, increased immune cell infiltration, reduced neuronal and oligodendrocyte survival, and impaired locomotor recovery [74].

These findings indicate that suppressing glial responses is not necessarily beneficial. However, evidence obtained in spinal cord injury (SCI) models cannot be extrapolated directly to chronically implanted neural devices, where persistent glial encapsulation and associated tissue changes may compromise electrode performance [23]. Accordingly, reductions in GFAP or Iba1 immunoreactivity around an implant should be interpreted alongside measures of tissue integrity, neuronal survival, and, where possible, functional outcomes. The desired glial response therefore depends on the biological context, the intended function of the biomaterial, and the time elapsed after injury or implantation.

### 3.4. Neuronal Sensing of the Material–Tissue Interface

Neurons sense the properties of the material–tissue interface through adhesion complexes, mechanosensitive systems, the cytoskeleton, and growth cones. In contrast to astrocytes and microglia, whose responses to materials are primarily associated with changes in homeostatic, reactive, or immune functions, neuronal responses manifest in polarization, neurite growth dynamics, and the selection of axonal growth direction. Thus, the properties of the interface can influence not only neuronal attachment and survival but also the spatial organization of neurites.

The influence of topography is evident from the early stages of neuronal polarization. Micropillar topography oriented N-cadherin and the Golgi–centrosome complex toward the topographical contacts, with the first neurite preferentially emerging in the same direction and the subsequent axon extending along the pillars [75]. These findings demonstrate that surface topography can determine the direction of neuronal development even before the formation of a mature axon.

At the level of the growing axon, the physical and biochemical properties of the interface are sensed by the growth cone through dynamic adhesion complexes. Interactions with a permissive ECM were accompanied by the formation of integrin-associated point contacts containing focal adhesion kinase (FAK). Local FAK signaling regulated protrusion stabilization and growth cone turning in response to the spatial distribution of the ECM, whereas disruption of FAK-dependent signaling altered axon guidance [76]. Thus, the adhesion machinery of the growth cone enables local properties of the extracellular interface to be translated into directed axonal movement.

The mechanical state of the substrate can also directly regulate the dynamics of neuronal processes. Axons of Xenopus retinal ganglion cells responded to spatial variations in the mechanical properties of their environment, preferentially growing toward softer regions. This response was associated with mechanosensitive ion channels and changes in intracellular Ca^2+^, while pharmacological disruption of mechanosensitive signaling suppressed mechanically guided axonal growth [77]. These findings demonstrate that the mechanical properties of the interface can themselves serve as a signal that is transduced by neurons into changes in growth cone behavior.

## 4. Intracellular Transduction of Material–Tissue Interface Signals

**Figure 4:**
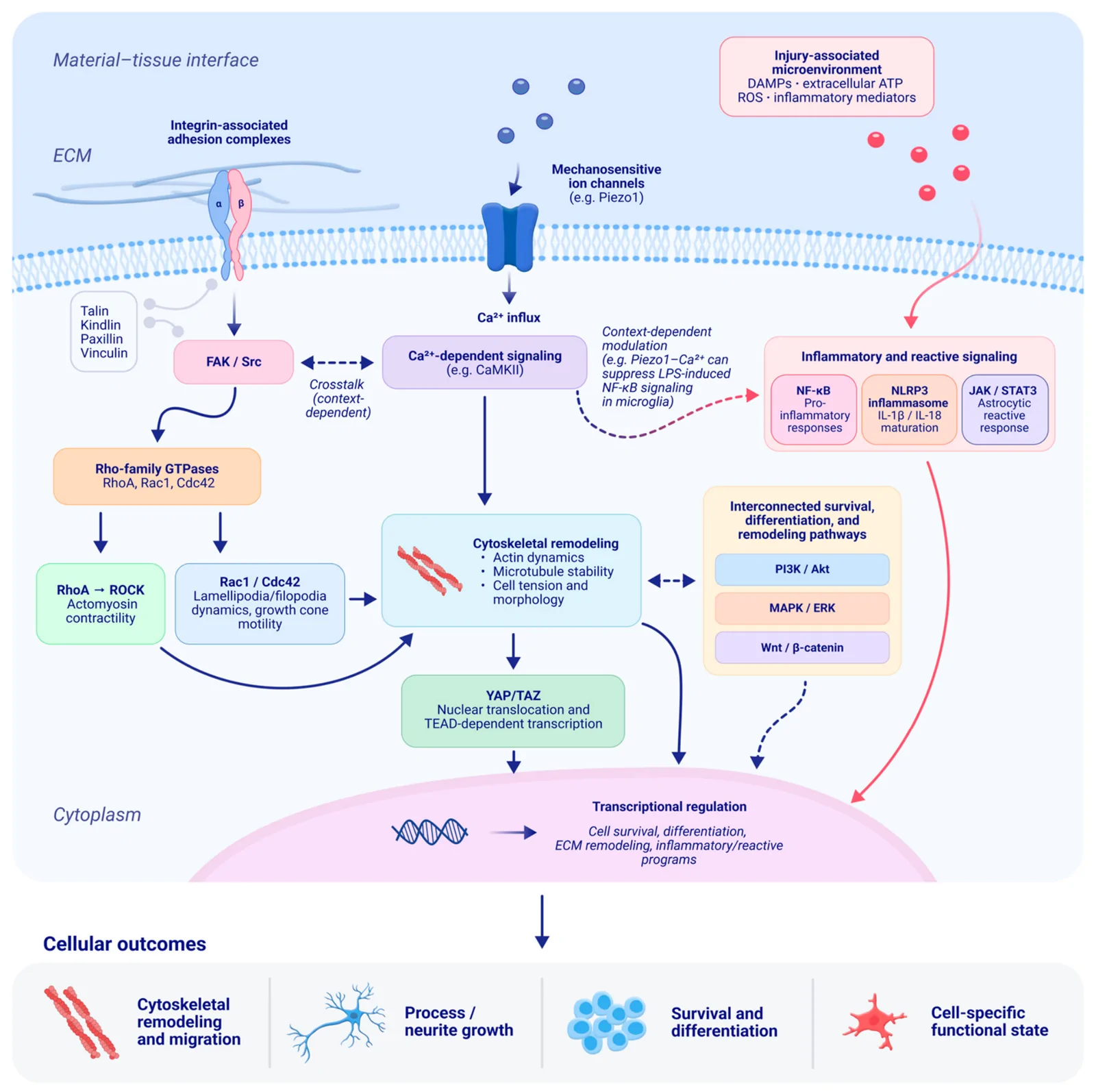
Overview of intracellular signaling pathways integrating material–tissue interface signals in neural cells. Material–tissue interface cues are sensed through ECM-engaged integrin-associated adhesion complexes and mechanosensitive ion channels such as Piezo1. Integrin-associated signaling involves FAK/Src and Rho-family GTPases (RhoA, Rac1, and Cdc42), whereas Piezo1 couples mechanical stimuli to Ca^2+^-dependent signaling. These sensing systems interact with cytoskeletal and transcriptional regulators, including YAP/TAZ, and with interconnected PI3K/Akt, MAPK/ERK, and Wnt/β-catenin signaling pathways that regulate cell survival, differentiation, and structural remodeling. In parallel, an injury-associated microenvironment comprising DAMPs, extracellular ATP, ROS, and inflammatory mediators engages inflammatory and reactive signaling involving NF-κB, inflammasome activation, and JAK/STAT3. Dashed arrows indicate context-dependent or indirect regulatory relationships, including modulation of inflammatory signaling by Piezo1-dependent Ca^2+^ influx, whereas double-headed dashed arrows represent potential bidirectional interactions between signaling systems, such as FAK/Src and Ca^2+^-dependent signaling. Together, these interconnected pathways regulate cytoskeletal remodeling, transcriptional programs, and cell-type-dependent functional outcomes. Solid arrows indicate the direction of signaling or downstream cellular responses, whereas red arrows highlight signaling associated with the injury microenvironment. Box colors group functionally related components and do not encode additional information.

Following recognition of the material–tissue interface, information about its adhesive, mechanical, and molecular properties is converted into intracellular signals that regulate cytoskeletal organization, migration, process growth, cell survival, and functional state (Figure 4). This process is mediated by several interconnected systems, including integrin-associated adhesion complexes, mechanosensitive ion channels, and signaling cascades that regulate transcription and cellular metabolism. The relationship between interface properties and cellular responses should therefore not be viewed as a sequence of independent linear pathways. The same components, including FAK, Ca^2+^-dependent signaling, and the cytoskeleton, can integrate signals received through different mechanisms of cellular sensing.

### 4.1. Integrin-Dependent Mechanotransduction: FAK/Src, RhoA/ROCK, and YAP/TAZ

Integrins link the ECM to the cytoskeleton and provide one of the major pathways through which material properties are translated into intracellular responses. Following ligand binding, integrins assemble complexes containing talin, kindlin, paxillin, vinculin, FAK, and other adhesome proteins, through which actin cytoskeletal organization and intracellular signaling are regulated [35,39,78,79]. FAK occupies a central position within this system. Its autophosphorylation at Tyr397 creates a binding site for Src-family kinases and promotes the formation of the FAK–Src signaling complex, linking integrin engagement to multiple downstream pathways [79]. The organization of these complexes, however, is cell-type dependent. Neurons are characterized by dynamic integrin-associated adhesions within the growth cone rather than the typical mature focal adhesions described in many other adherent cell types [80].

Functional specialization among individual components of this complex has been directly demonstrated in neurons. Integrins, talin, and FAK were required for neuronal process outgrowth, whereas vinculin was dispensable for neurite outgrowth but specifically required for neuronal mechanosensing: following vinculin depletion, growth cones lost their ability to distinguish between soft and stiff substrates [80]. Growth cones generated dynamic stresses of approximately 10–12 Pa, while integrin-associated point contacts were localized predominantly at the base of filopodia. Thus, individual components of the adhesion complex can perform distinct functions in supporting neurite growth and mechanosensing.

Mechanical signal transduction from adhesion complexes is closely coupled to remodeling of the actin cytoskeleton. Rho-family GTPases regulate the spatial organization of this process: RhoA/ROCK contributes to the control of actomyosin contractility, whereas Rac1 and Cdc42 regulate lamellipodia and filopodia formation and growth cone dynamics [81,82]. The cytoskeleton therefore acts both as an effector of mechanical signaling and as part of a mechanical feedback loop: forces generated by the actomyosin network alter the mechanical load on integrin-associated complexes and thereby influence the state of the adhesion machinery itself.

One mechanism by which mechanical signals are translated into changes in gene expression is YAP/TAZ-dependent signaling. YAP and TAZ respond to the state of the actin cytoskeleton, cellular tension, and the organization of adhesion complexes and, following translocation to the nucleus, regulate transcriptional programs primarily through TEAD-family transcription factors [83,84]. The relationship between focal adhesions and YAP was further resolved by perturbing FAK, talin, and vinculin function, which reduced mechanosensitive YAP nuclear localization through distinct mechanisms [85].

The relevance of this pathway has also been demonstrated in neural cells. A polyacrylamide matrix with dynamically tunable stiffness revealed a restricted temporal window during which the mechanical properties of the ECM determined the neurogenic differentiation of adult neural stem cells. Increasing YAP expression during this period suppressed neurogenesis, whereas YAP knockdown enhanced it, with this effect being linked to the interaction between YAP and β-catenin [86]. Thus, mechanical signals from the ECM can influence not only immediate cell morphology but also subsequent cell fate decisions.

Integrin-associated mechanotransduction, however, does not operate in isolation from other mechanosensing systems. In DRG neurons, stiffness-dependent axon regrowth was regulated by Piezo1 through the Ca^2+^–CaMKII–FAK–actin signaling cascade. Piezo1 was localized to the growth cone, and modulation of its activity affected actin retrograde flow and the neuronal response to substrate stiffness. Piezo1 knockdown in adult DRG neurons enhanced axon regeneration following sciatic nerve injury [87]. These findings indicate that FAK can participate not only in signal transduction downstream of integrin-associated complexes but also in the integration of mechanical signals transmitted through mechanosensitive ion channels, linking adhesion-dependent and ion channel-mediated mechanotransduction.

### 4.2. Mechanosensitive Ion Channels and Ca^2+^ Signaling

Mechanosensitive ion channels provide another route through which mechanical properties of the cellular microenvironment are converted into intracellular signals. Among these channels, Piezo1 is one of the best-characterized mediators of mechanotransduction and functions as a mechanically activated nonselective cation channel that couples mechanical stimuli to ion flux and Ca^2+^-dependent signaling [88,89]. In human neural stem/progenitor cells, Piezo1 mediated mechanically induced ionic currents, while cell-generated traction forces elicited channel-dependent Ca^2+^ transients whose frequency increased with substrate stiffness. Pharmacological inhibition of the channel or siRNA-mediated Piezo1 knockdown suppressed neurogenesis and enhanced astrogenesis, while Piezo1 knockdown also reduced YAP nuclear localization [90].

In astrocytes, Piezo1 was detected in cellular processes and mediated mechanically evoked Ca^2+^ responses and subsequent ATP release [45]. Astrocytic Piezo1 deletion abolished ATP-dependent potentiation of neural stem cell proliferation in vitro and impaired adult hippocampal neurogenesis in vivo, whereas exogenous ATP partially rescued this effect [45]. These findings establish a functional connection between astrocytic mechanosensing and the regulation of neighboring cell populations through a soluble mediator. Unlike studies limited to intracellular Ca^2+^ responses, this work provides evidence that mechanical signals detected by astrocytes can influence neurogenic processes beyond the mechanically stimulated cell.

Piezo1 also participates in Ca^2+^-dependent regulation of inflammatory signaling in microglia. In BV2 cells and primary microglia, channel activation increased intracellular Ca^2+^ while suppressing LPS-induced NF-κB signaling and the production of TNF-α and IL-6 [71]. This response illustrates that activation of a mechanosensitive channel does not necessarily correspond to an increase in inflammatory activity. The interaction between mechanotransduction and inflammatory signaling is examined further in Section 4.4.

Mechanosensitive ion channels operate alongside adhesion-dependent signaling systems, providing interconnected routes for translating mechanical cues into cellular responses. Piezo1-dependent Ca^2+^ signaling can interact with downstream regulatory networks involving YAP and other effectors, while integrin-associated pathways contribute to cytoskeletal remodeling and mechanosensitive transcriptional regulation. Their combined activity links the mechanical state of the extracellular environment to processes governing cell survival, differentiation, and structural remodeling, discussed in the following section.

### 4.3. Survival, Differentiation, and Remodeling Pathways

Initial recognition of the material–tissue interface through adhesion complexes and mechanosensitive channels is subsequently coupled to signaling pathways that regulate cell survival, differentiation, and structural remodeling. These processes do not constitute independent linear cascades. FAK/Src, Rho-associated cytoskeletal signaling, and Ca^2+^-dependent pathways interact with PI3K/Akt, MAPK/ERK, YAP/TAZ, and Wnt/β-catenin signaling; consequently, a single physical or biochemical material cue can simultaneously alter multiple components of the cellular response [78,79,83,90,91].

Mechanotransduction influences cell fate through interactions between YAP/TAZ and other fate-regulating pathways. In neural stem cells, Angiomotin (AMOT) participates in coupling ROCK-dependent cytoskeletal signaling to YAP and β-catenin during mechanosensitive differentiation [92]. Modulation of the AMOT–YAP interaction affected β-catenin activity and neuronal differentiation, demonstrating that mechanical signals are transduced through interactions among multiple intracellular regulatory systems rather than through isolated activation of YAP/TAZ [92].

This interconnection is also evident in biomaterial systems in which the properties of the material itself change over time. Culture of human neural stem cells on an alginate hydrogel promoted astrocytic differentiation and was accompanied by F-actin reorganization, Piezo1-dependent Ca^2+^ signaling, and YAP nuclear translocation [93]. Inhibition of Piezo1 or YAP/TEAD-dependent transcription reduced astrocytic differentiation. In this system, changes in the mechanical properties of the hydrogel were therefore associated with sequential engagement of Piezo1-dependent Ca^2+^ signaling, the cytoskeleton, and YAP-dependent transcription in the regulation of cell fate [93].

Another level of regulation involves PI3K/Akt signaling, which integrates signals from growth factor receptors, integrins, and other membrane-associated systems and contributes to the regulation of cell survival and differentiation. In a recent neural biomaterial model, lithium–calcium–silicate (LCS) microspheres were incorporated into bioprinted GelMA-based constructs containing neural stem cells [94]. The material promoted neuronal differentiation and maturation and was accompanied by increased levels of p-PI3K and p-Akt. PI3K inhibition with LY294002 attenuated both the downstream signaling changes and the increase in βIII-tubulin, supporting an association between PI3K/Akt signaling and the observed pro-neurogenic effect of the material [94].

Signaling systems that regulate cell fate can also be deliberately engaged through material functionalization. A composite hydrogel for spinal cord injury combined acellularized spinal cord matrix, aligned PCL microfibers, and WAY-316606, an indirect activator of canonical Wnt/β-catenin signaling [95]. In neural stem cell cultures, the system promoted neuronal differentiation and was accompanied by increased β-catenin and downstream TCF4/LEF1 signaling, while following implantation it promoted endogenous NSC recruitment and neural regeneration [95]. However, because ECM composition, architecture, and biochemical signaling were modified simultaneously, the overall effect cannot be attributed exclusively to the Wnt/β-catenin pathway. This example illustrates both the opportunities and the limitations of attempts to link complex biomaterial designs to individual intracellular mechanisms.

In astrocytes and microglia, these signaling pathways also interact with regulatory networks that control inflammatory and reactive responses, particularly under conditions of tissue injury and implantation.

### 4.4. Inflammatory and Reactive Signaling

Recognition of the material–tissue interface is coupled to signaling pathways that regulate inflammatory and reactive states in glial cells. Following implantation, these processes occur in the context of tissue injury and are shaped by a combination of DAMPs, extracellular ATP, ROS, and other local signals [23,96]. NF-κB is one of the central regulators of the inflammatory response, linking the activation of pattern-recognition and cytokine receptors to the expression of pro-inflammatory mediators and components of the NLRP3 inflammasome [97]. Its activation at the implantation site reflects the combined influence of material-associated cues and the surrounding injury-related microenvironment.

As discussed in Section 4.2, Piezo1-dependent signaling can modulate microglial inflammatory responses [71], illustrating the interaction between mechanotransduction and inflammatory regulatory pathways.

In astrocytes, JAK/STAT3 signaling is one of the key regulators of the reactive response. Selective disruption of STAT3 signaling in GFAP+ astrocytes following spinal cord injury reduced astrocyte hypertrophy and impaired astroglial scar organization, while increasing the spread of inflammatory cells and the extent of tissue damage [72]. Astrocyte reactivity should therefore not be regarded as an unequivocally pathological process; rather, its functional consequences depend on the nature and spatiotemporal organization of the response.

Inflammasome signaling has also been investigated at the microelectrode–tissue interface following implantation of a Utah electrode array into the rat somatosensory cortex [98]. Elevated expression of several inflammasome sensors, including NLRP1, NLRP3, NLRC4, and AIM2, as well as ASC and caspase-1, persisted at the implantation site. These changes were accompanied by increased IL-1β and IL-18 levels and evidence of gasdermin D-associated pyroptosis throughout the observation period, encompassing both acute and chronic phases [98]. This study provides evidence of sustained innate immune signaling at an implanted neural interface.

Changes in inflammatory and reactive signaling can alter the functional state of glial cells and the mediators they release into the surrounding tissue. Whether these changes subsequently affect neighboring neural cells requires evidence of intercellular communication beyond the activation of intracellular pathways.

### 4.5. From Intracellular Mechanotransduction to Intercellular Signaling

The signaling pathways discussed above have been studied primarily as intracellular responses to material properties. Whether they are relevant to neuron–glia interactions depends on whether such responses are transmitted to neighboring cells. Three principal routes can be distinguished: (i) the release of soluble mediators, including ATP, cytokines, and trophic factors; (ii) changes in glial homeostatic functions, such as glutamate uptake and extracellular K^+^ regulation; and (iii) ECM deposition and remodeling or extracellular vesicle secretion. These processes provide potential links between material-induced intracellular signaling and changes in the neural microenvironment.

Several studies have shown that biomaterial properties influence these cellular functions. Substrate stiffness affected astrocytic glutamate uptake and ROS production [47], and substrates of different stiffnesses altered the proportion of astrocytes expressing ECM-associated proteoglycans, including aggrecan, brevican, and neurocan [61]. Increased stiffness also enhanced inflammatory responses in astrocytes and microglia, with stiffer regions of implanted composites eliciting more pronounced foreign body reactions in the rat brain [99]. Microgrooved substrates increased astrocytic Ca^2+^ signaling and ATP release, linking a topographical cue directly to the secretion of an extracellular signaling molecule [100]. These observations, however, do not in themselves establish how the resulting changes affect neighboring neurons or glial cells.

Other biomaterial-based studies have examined responses in neighboring neural cells. Graphene oxide enhanced astrocytic inward-rectifying K^+^ channel activity and glutamate uptake, while subsequent co-culture experiments revealed changes in neuronal excitability and inhibitory synaptic transmission [54]. On patterned laminin substrates, astrocyte-associated fibronectin contributed to neurite guidance, providing evidence for an ECM-mediated interaction [60]. Similarly, three-dimensional graphene foam reduced LPS-induced TNF-α and IL-1β release from microglia, and conditioned medium from these cultures increased neural stem cell viability [59]. An IGF-1-bioactive hydrogel also modified the miRNA cargo of extracellular vesicles released by neural stem cells and their progeny, with the resulting vesicles exhibiting biological activity in experimental models [101]. Although these studies demonstrate material-associated changes in cellular communication or its potential mediators, the intracellular pathways responsible and the causal contribution of individual extracellular signals were not consistently established.

Distinguishing intracellular responses from intercellular signaling is essential when interpreting mechanotransduction studies. Studies of neural stem cells cultured on alginate hydrogels have implicated mechanosensitive ion channels and YAP signaling in astrocytic differentiation [93], while substrate stress relaxation has been linked to myosin II- and FAK-dependent regulation of neural stem cell fate [102]. These findings establish roles for intracellular mechanotransduction in cell-intrinsic responses but do not demonstrate that the same pathways mediate material-induced signaling between neurons and glia. Additional mechanistic evidence from non-biomaterial experimental systems helps identify potential links between mechanotransduction, glial responses, and intercellular signaling. Astrocytic Piezo1 regulates hippocampal neurogenesis through mechanically induced Ca^2+^ signaling and ATP release [45], and pharmacological activation of astrocytic Piezo1 has been associated with suppressed LPS-induced release of inflammatory mediators [103]. In microglia, Piezo1 activation suppresses LPS-induced TNF-α and IL-6 production through Ca^2+^-dependent regulation of NF-κB signaling [71]. Separately, microglia-derived IL-1α, TNF, and C1q can jointly induce a reactive astrocyte state associated with neurotoxic activity [20]. Together, these findings identify plausible routes through which mechanosensitive signaling could influence neuron–glia interactions, although their involvement in responses to specific biomaterial properties remains to be demonstrated.

Overall, the available evidence supports distinct components of the proposed sequence linking material properties, intracellular signaling, glial functional states, and responses in neighboring neural cells, but it rarely captures the entire sequence within a single experimental system. Establishing such causal links will require pathway-specific interventions combined with measurements of extracellular mediators, glial homeostatic functions, and neuronal outcomes. Blocking or restoring candidate mediators would help distinguish direct material effects from glia-mediated signaling and determine whether the identified intracellular pathways are required for the observed intercellular responses.

## 5. Material Properties as Design Variables

Material properties define the physical and chemical conditions under which the material–tissue interface is formed. The preceding sections addressed the interfacial protein layer, cellular recognition, and intracellular signaling pathways. Here, the focus shifts to material design parameters, including stiffness, architecture, surface chemistry, electrical conductivity, and degradation. These parameters are important not in isolation, but because their modulation provides a means of directing cellular responses.

### 5.1. Mechanical Properties—Stiffness, Viscoelasticity, Stress Relaxation, and Nonlinear Mechanics

Neural tissue is an ultrasoft, mechanically heterogeneous, and viscoelastic environment, and its mechanical properties therefore cannot be adequately described by a single elastic modulus. Measured parameters depend substantially on anatomical region, spatial scale, deformation mode and rate, and measurement method [104,105]. The spinal cord likewise exhibits region-dependent heterogeneity in its mechanical response. Dynamic mechanical analysis of the cervical, thoracic, and lumbar regions showed that the complex modulus increased nonlinearly with loading frequency, with the thoracic region exhibiting greater stiffness than the cervical and lumbar regions [106]. In a 2025 indentation study, the apparent moduli of porcine spinal cord white and gray matter were 128.7 and 403.8 Pa, respectively; the mechanical response was loading-rate dependent and exhibited pronounced stress relaxation [107]. However, absolute values of mechanical parameters vary considerably among studies. A systematic review of spinal cord mechanics identified substantial heterogeneity in experimental protocols, animal species, and measurement conditions, which currently precludes the establishment of a single reference range for the human spinal cord [108].

For biomaterial design, this means that mechanical matching should not be reduced to reproducing a single stiffness value. Regional differences in the mechanical properties of the brain and spinal cord, together with anisotropy in the cervical spinal cord, were used to guide the tuning of collagen-, fibrin-, and alginate-based hydrogels toward the dynamic mechanical characteristics of different CNS regions [109]. Current data on brain mechanics likewise demonstrate a pronounced dependence of the mechanical response on loading timescale and deformation regime, including nonlinear behavior and stress relaxation [104,105,110]. Relevant material parameters therefore extend beyond the elastic modulus to include viscoelasticity, stress-relaxation rate, nonlinearity, anisotropy, and spatial heterogeneity of mechanical properties.

The biological relevance of these parameters has been directly demonstrated in neural cell models. Stiffness-dependent changes in neural stem cell differentiation have been reported [111,112]. Cells, however, respond not only to absolute stiffness but also to its spatial distribution. In collagen hydrogels with stiffness gradients ranging from approximately 1.5 to 7 kPa, the orientation of NG108-15 neural cells depended on the combination of local stiffness and gradient magnitude [113]. In hyaluronic acid scaffolds with an aligned porous architecture and stiffnesses ranging from 0.8 to 3 kPa, softer Coll-IV/Fn-functionalized scaffolds enhanced axonal outgrowth ex vivo and altered primary astrocyte responses, including stiffness-dependent IL-10 production [114]. This latter example also illustrates that the observed cellular response cannot be unequivocally attributed to stiffness alone, because material composition and architecture varied simultaneously.

Viscoelasticity represents an independent regulator of cellular responses. In polyacrylamide substrates with comparable initial stiffness, Qiao et al. independently varied stress relaxation by introducing reversible DNA-based crosslinks, obtaining characteristic relaxation times of approximately 60–175 s. Greater stress relaxation shifted neural stem cell fate commitment toward astrocytic differentiation and altered intracellular RhoA dynamics. Notably, the average period of RhoA activity oscillations decreased from approximately 17 min on non-relaxing substrates to 5 min on stress-relaxing substrates. Pharmacological inhibition of myosin II or FAK attenuated the relaxation-dependent increase in astrocytic differentiation, implicating actomyosin contractility and focal adhesion signaling in the cellular response [102]. In a 3D system, hydrogels with stress-relaxation half-times spanning approximately 10^2^–10^5^ s were used to investigate neural progenitor cell responses. Rapidly relaxing hydrogels (approximately 10^2^–10^3^ s) with low network connectivity supported NPC spreading and proliferation while preserving stemness and subsequent differentiation potential, whereas slowly relaxing networks restricted cell spreading and reduced proliferation and differentiation capacity [115]. Thus, identical initial substrate stiffness does not necessarily result in an equivalent cellular response when the temporal mechanical behavior and capacity for local matrix remodeling differ.

A separate direction in mechanical design involves architecturally programmable bottlebrush polymer networks that enable independent control over initial softness and nonlinear strain stiffening [116]. Building on this approach, injectable bottlebrush hydrogels have been developed that combine a low Young’s modulus, pronounced strain stiffening, and thermally induced gelation at physiological temperature; in preclinical testing, these materials exhibited low cytotoxicity and a moderate tissue response in vivo [117]. More recent bottlebrush networks maintain a modulus below 1 kPa at high swelling ratios and can withstand substantial deformation [118]. These systems expand the range of independently tunable mechanical parameters; however, direct evidence of their effects on neuron–glia interactions and integration with neural tissue remains limited.

Mechanical matching of biomaterials extends beyond stiffness alone and requires consideration of viscoelasticity, stress relaxation, nonlinearity, anisotropy, and spatial heterogeneity in relation to the mechanics of the target neural tissue. Given the lack of a universal mechanical reference and the sensitivity of cellular responses to time-dependent mechanics, a single “optimal” stiffness value is unlikely to provide an adequate design criterion for neural interfaces.

### 5.2. Architecture and Topography—Fiber Alignment, Anisotropy, Pore Organization, and Surface Porosity

Material architecture defines the spatial conditions under which cells interact with the interface through several parameters, including the nanotopography of individual fiber surfaces, fiber orientation, packing density, pore connectivity, and the organization of channels within the scaffold. Anisotropy is particularly relevant to neural tissue. The white matter of the spinal cord is organized into longitudinally oriented axonal tracts; accordingly, aligned fibers, longitudinal channels, and oriented pores are used to reproduce the directional architecture of the tissue and guide the trajectories of regenerating processes [119]. However, implant architecture cannot be considered independently of interface chemistry and mechanics, because changes in geometry simultaneously alter the contact area, accessibility of adhesive sites, and the mechanical conditions of cell–material interactions.

The influence of fibrous architecture has been directly demonstrated in neural progenitor cells and neurons. On electrospun collagen nanofibers, proliferation of spinal cord-derived NPCs was higher than on collagen-coated planar surfaces, and this effect was dependent on β1-integrin and MEK/ERK signaling [120]. Fiber diameter also altered cellular responses in DRG explants cultured on aligned poly-L-lactic acid (PLLA) fibers. On fibers with a diameter of 293 ± 65 nm, neurite length was 42% and 36% lower than on fibers with diameters of 759 ± 179 and 1325 ± 383 nm, respectively, whereas the pattern of Schwann cell migration did not fully parallel the changes in neurite outgrowth [121]. Even when fiber orientation is held constant, fiber diameter can therefore differentially affect distinct cell populations.

Fiber alignment alone, however, does not determine the direction of neurite growth. In DRG explants cultured on uniaxially aligned electrospun nanofibers, neurites could extend either parallel or perpendicular to the fiber direction. The pattern of contact guidance depended on fiber density, laminin coating, and the properties of the underlying substrate. At low fiber density, neurite growth occurred predominantly along the fiber direction, whereas increasing fiber density under certain conditions resulted in perpendicular neurite orientation. Laminin coating could shift the direction of growth back toward parallel alignment [122]. Topographical guidance therefore emerges from the combination of geometric and adhesive cues rather than being determined by fiber alignment alone.

In 3D scaffolds, bulk porosity becomes an additional important architectural parameter. Pore size, connectivity, and orientation determine the space available for cell infiltration and migration, process extension, and the transport of oxygen, nutrients, and soluble factors. In porous GelMA hydrogels with a mean pore size of 168 ± 71 μm and a porosity of 77.1%, an interconnected architecture enhanced neural stem cell migration and neuronal differentiation compared with nonporous GelMA [123]. Directionally organized pores and channels are particularly relevant to SCI. Anisotropic scaffolds containing longitudinal fibers, channels, or pores can provide spatial guidance for axonal growth across the lesion; however, the architecture must simultaneously permit cellular infiltration while maintaining the required structural organization of the scaffold [119].

The surface architecture of individual fibers represents another important design parameter because it alters the effective surface area and influences not only direct cell–material interactions but also the organization of the adsorbed protein layer. In electrospun poly(D,L-lactide) fibers, the formation of surface nanopores increased the calculated surface area by 62% and was accompanied by an 80% increase in protein adsorption from fetal bovine serum compared with nonporous fibers, as well as greater initial cell adhesion [124]. In neural cell models, both the presence and geometry of surface nanoporosity are relevant. On aligned PLLA fibers, larger nanopores promoted neural stem cell proliferation and differentiation, whereas smaller nanopores were more favorable for migration. However, changes in nanopore size were accompanied by changes in surface roughness, wettability, and crystallinity, precluding the isolation of nanoporosity as the sole cause of the cellular effects [125]. In electrospun PLA/PCL scaffolds, the use of DCM/THF resulted in the formation of surface-localized pores, with a surface porosity of 27% and a mean pore depth of 270 ± 50 nm. In primary cortical neuron–glia cultures, these scaffolds maintained approximately 80% cell viability over 10 days and increased neuronal density compared with smooth fibers and the control, while astrocytes exhibited a more highly branched and less rounded morphology than on HFIP-derived scaffolds [63]. Surface nanoporosity can therefore be considered a distinct architectural parameter of the interface that can alter protein adsorption and cellular responses, although its effects depend on accompanying changes in surface chemistry and physical properties.

Architectural features of a material may also be sensed differently by distinct cell populations. On anodized alumina surfaces, changes in nanopore diameter and spacing were associated with differences in primary astrocyte adhesion and GFAP immunoreactivity [62]. Together with findings in neurons, NPCs, and Schwann cells, this indicates that biomaterial architecture cannot be optimized on the basis of a single cell type. Fiber size and orientation, bulk porosity, and surface nanoporosity form an interconnected set of topographical cues whose effects are cell-type dependent and are further modulated by the chemical and mechanical properties of the interface.

### 5.3. Surface Chemistry and Ligand Presentation—From Cell Adhesion to Antifouling Interfaces

Surface chemistry defines the molecular-level interactions of a material with proteins and cellular receptors. Hydrophilicity, surface charge, and the chemical structure of functional groups influence protein adsorption and conformation, whereas immobilization of ECM-derived ligands enables direct modulation of receptor-mediated adhesion [33,35,37,38]. Cellular responses depend not only on the chemical identity of a ligand but also on its density, spatial organization, and physical accessibility. Chemical design of biomaterials spans a continuum from bioadhesive surfaces that promote specific cell–material interactions to antifouling interfaces that limit nonspecific protein adsorption and cell attachment.

The importance of the physical presentation of adhesive ligands has been demonstrated using supported lipid bilayers with tunable lateral mobility of surface molecules. At comparable chemical composition, restricting ligand mobility enhanced integrin clustering, focal adhesion formation, FAK–MEK/ERK signaling, and neuronal differentiation of neural stem cells, whereas high ligand mobility was associated with reduced adhesion and preferential astrocytic differentiation [126]. Cellular responses thus depend not only on the presence of an adhesive motif but also on the physical context in which it is presented at the substrate surface. The density of bioactive epitopes represents an additional design variable. In self-assembling peptide amphiphile nanofibers, a high density of the laminin-derived IKVAV epitope enhanced neuronal differentiation of neural progenitor cells while reducing astrocytic differentiation [127]. The response also depends on the combination of ECM components. Combined collagen IV and fibronectin promoted neurite outgrowth more strongly than either component alone [114]. Biofunctionalization is therefore better understood not simply as a means of increasing overall surface adhesiveness, but as a strategy for controlling the identity, density, and mode of presentation of molecular cues.

For chronic neural interfaces, the design objective may instead be to limit nonspecific interactions between the surface and the biological environment. One approach involves zwitterionic polymer coatings, whose highly hydrated character limits nonspecific protein adsorption and subsequent cell attachment. Silicon neural probes were modified with a poly(sulfobetaine methacrylate)/polydopamine coating. The coating reduced protein adsorption by 89% and fibroblast adhesion by 86% relative to uncoated silicon. One week after intracortical implantation, coated probes exhibited reduced GFAP-associated astrocytic responses, Iba1/lectin-associated microglial responses, and IgG leakage around the implant [128]. Antifouling surface modification can affect both the initial interaction with proteins and the early tissue response to a neural implant.

In a subsequent study, poly(sulfobetaine methacrylate) (PSBMA) was covalently grafted directly onto silicon neural probes, providing coating stability and antifouling properties for at least four weeks in vitro. The coating reduced protein adsorption and the attachment of fibroblasts and microglia. Two-photon imaging performed in vivo at 2 and 6 h after intracortical implantation showed that PSBMA-coated probes had an approximately threefold reduction in the surface area covered by microglial processes compared with control probes. This effect was primarily associated with reductions in the area and volume of microglial end-feet contacting the surface, whereas their number per unit area did not change substantially [129]. The antifouling interface therefore did not prevent the directed microglial response to implantation-associated injury, but limited the subsequent spreading of microglial processes directly along the material surface.

Regenerative scaffolds require controlled cell–material interactions that support adhesion, migration, and process growth, whereas chronic neural interfaces may benefit from reduced nonspecific protein adsorption and glial attachment. A promising strategy is to combine a low-fouling background with the selective presentation of bioactive ligands. The balance between adhesion and antifouling can thus be treated as a tunable property of the material–tissue interface, with the appropriate balance depending on material function, the target cell population, and the duration of interaction with neural tissue.

### 5.4. Electrical Conductivity and Electroactive Properties

The electrical properties of a material provide an additional level of regulation at the material–tissue interface because neural tissue function depends on cellular electrical activity and the propagation of bioelectrical signals. In biomaterials, a distinction should be made between electrical conductivity, which enables charge transport through the material, and electroactivity, whereby an electrical stimulus is generated or transmitted in response to an external input. Conductivity alone, however, is not sufficient to establish a biological effect. Incorporation of conductive components can simultaneously alter mechanics, topography, surface charge, and other interfacial properties. The causal contribution of electrical properties can therefore be assessed most convincingly in systems in which a conductive scaffold is investigated in combination with controlled electrical stimulation.

This approach was implemented using three-dimensional PEDOT/chitosan/gelatin scaffolds containing neural stem cells [130]. Electrical stimulation at 50 mV for 4 h/day enhanced neuronal differentiation of NSCs, whereas pharmacological inhibition of voltage-gated Ca^2+^ channels attenuated this effect. In this system, the conductive scaffold served as a medium for transmitting the electrical stimulus, while the cellular response was associated with Ca^2+^-dependent signaling.

Beyond their electrical properties, PEDOT-based materials are also of interest because of their potential cytocompatibility and influence on cellular responses to electrical stimulation. Microstructured PEDOT:PSS electrodes have demonstrated cytocompatibility in vitro, supporting their use as interfaces for cell culture and bioelectronic applications [131]. Moreover, PEDOT:PSS-coated electrodes have been shown to reduce mitochondrial superoxide generation and associated cellular damage during pulsed electric field exposure [132]. These findings suggest that PEDOT-based interfaces may contribute to the modulation of electrically induced oxidative stress in addition to facilitating charge transfer. However, these effects are dependent on the material formulation and stimulation conditions, and their relevance to long-term neuron–glia interactions requires further investigation.

The combination of electrical conductivity with directional architecture was implemented using aligned CNT/GelMA hydrogel fibers. The material simultaneously reproduced a fibrous architecture and enabled transmission of an electrical stimulus. Aligned CNT/GelMA fibers supported oriented adhesion of PC12 cells, while their combination with electrical stimulation enhanced neuronal differentiation of NSCs. In a rat model of SCI, the combination of a conductive aligned scaffold and electrical stimulation was associated with enhanced axonal regeneration and remyelination, as well as improved electrophysiological and motor outcomes [133]. This system illustrates how electrical and architectural cues can act in combination, while also precluding attribution of the observed effects to conductivity alone because fiber alignment is itself a biologically active design parameter.

A different class of electroactive biomaterials can locally generate, rather than merely conduct, an electrical signal. A biodegradable 3D piezoelectric scaffold was developed to convert ultrasound into local electrical stimulation [134]. In a rat model of complete spinal cord transection, ultrasound-driven wireless stimulation through the implanted scaffold was associated with enhanced axonal regeneration and remyelination and improved motor recovery. This approach differs fundamentally from passive conductive materials because the piezoelectric scaffold simultaneously provides structural support and converts the mechanical energy of an external stimulus into a local electrical signal.

In a subsequent study, this principle was implemented in an injectable electrogenic hydrogel containing black phosphorus nanoplates. When exposed to a rotating magnetic field, the material generated electrical signals, while wireless electrical stimulation enhanced neuronal differentiation of NSCs and was associated with activation of PI3K/Akt signaling. In a mouse model of complete spinal cord transection, treatment with this system attenuated the inflammatory response and was accompanied by increased neurogenesis and synapse formation, as well as functional recovery [135]. Importantly, the material combined mechanical, conductive, electrogenic, and immunomodulatory properties, precluding attribution of the observed in vivo effects exclusively to electrical stimulation.

Electrical conductivity and electroactivity are functional material properties that enable electrical signals to be transmitted or locally generated at the material–tissue interface. The strongest available evidence indicates that such signals can influence neuronal differentiation, axonal growth, and other components of the cellular response. However, electrical properties are almost invariably coupled to changes in material mechanics, architecture, and chemistry, making experimental isolation of their biological contribution essential. For chronic neural interfaces, additional considerations include electrochemical stability, impedance, long-term recording and stimulation stability, and sustained tissue integration.

### 5.5. Degradation and Temporal Evolution of Material Properties

For degradable biomaterials, degradation represents a dynamic parameter of the interface because material breakdown is accompanied by concurrent changes in mechanical properties, architecture, surface area, and the chemical environment of surrounding cells. The relevant consideration is therefore not simply whether a material is ultimately resorbed, but whether its degradation rate is matched to the timescales of cellular infiltration, process growth, and new tissue formation. Excessively rapid loss of structural integrity may eliminate the scaffold’s guidance and mechanical functions before regeneration is complete. For example, in multilumen alginate scaffolds developed for spinal cord repair, the shear modulus in vitro decreased from 155 to 5 kPa within 2 days, despite preservation of the external scaffold geometry for more than 28 days. Following implantation in a complete spinal cord transection model, the scaffolds degraded faster than axons could grow across the 2-mm lesion, thereby limiting their guidance function [136].

Conversely, prolonged structural persistence is not necessarily advantageous. As discussed above, urinary bladder matrix-derived ECM hydrogels with slower degradation showed less extensive replacement by newly formed tissue than more rapidly degrading formulations in the post-stroke brain [43]. Thus, for a regenerative interface, degradation should provide a sufficient temporal window for the material to perform its intended function, followed by progressive clearance that permits tissue ingrowth and remodeling.

The relationship between degradation rate and regeneration is not linear. In an SCI model, injectable spinal cord- and urinary bladder-derived ECM hydrogels supported cellular infiltration and axonal and vascular ingrowth but rapidly lost structural integrity after implantation while the cystic cavity continued to develop [137]. Degradation in a regenerative interface must be coordinated with the rates of tissue ingrowth and remodeling rather than simply maximized or minimized.

For synthetic polymers, the chemistry of degradation products represents an additional consideration. Hydrolytic degradation of PLA, PGA, and PLGA generates low-molecular-weight products, including lactic and glycolic acids, whose accumulation can alter local pH. This effect has been particularly well characterized for PLGA. Pronounced pH gradients have been experimentally detected within degrading PLGA microspheres, with local values as low as pH 1.5 [138]. The rate at which acidic degradation products are generated and accumulate depends not only on degradation time but also on molecular weight, copolymer composition, crystallinity, monomer sequence, geometry, and the transport of water and hydrolysis products within the material [138,139].

Degradation kinetics are determined not only by polymer chemistry but also by material composition, architecture, and the local microenvironment. Polymer composition, fiber morphology, and environmental conditions can jointly shape degradation behavior. For example, PLA/PCL electrospun scaffolds containing porous DCM/THF-derived fibers exhibited more pronounced signs of degradation than scaffolds containing smooth HFIP-derived fibers, with these differences becoming more pronounced under oxidative Fenton conditions [63]. These surface changes were not accompanied by a proportional loss of mass. Degradation behavior should therefore be characterized at multiple levels, including mass loss, architectural changes, and surface chemistry, and assessed under conditions that account for the pathological microenvironment of injured tissue.

Degradation products should also not be considered solely in terms of their potential cytotoxicity. PLA hydrolysis ultimately generates lactic acid, which at physiological pH is present predominantly as lactate [140]. Lactate is a physiological metabolite in neural tissue, and in vivo studies have demonstrated a concentration gradient between astrocytes and neurons that is consistent with intercellular lactate transfer [141,142]. However, there is currently insufficient direct evidence that lactate generated by the degradation of PLA-based neural scaffolds participates in these metabolic interactions or exerts beneficial effects on neuron–glia crosstalk.

The effects of biomaterial degradation on neural cells extend beyond the potential biological activity of individual degradation products. Degradation simultaneously alters multiple features of the interface, including the local concentration of hydrolysis products, pH, surface integrity, the size and abundance of material fragments, and mechanical properties. Each of these factors can potentially modify cellular responses, but a direct link between the degradation of a specific neural scaffold and a particular intracellular inflammatory pathway must be established experimentally.

## 6. Biomaterial Strategies for Neural Tissue Regeneration and Integration

### 6.1. Cell Delivery and Formation of a Regenerative Niche

Cell transplantation following neural tissue injury is limited not only by the retention of transplanted cells at the lesion site but also by their survival and functional integration within the adverse post-injury microenvironment. In such systems, biomaterials establish a local niche that simultaneously provides spatial localization of the graft and regulates cellular state. The relevance of this approach is supported by a systematic review and network meta-analysis of preclinical SCI models. Overall, combining neural stem/progenitor cells (NSPCs) with biomaterial scaffolds or hydrogels resulted in greater motor and histological recovery than cell therapy without a biomaterial, although the outcomes were characterized by substantial heterogeneity related to cell source, biomaterial type, SCI model, and experimental protocol [143].

The presence of a biomaterial alone, however, does not ensure graft survival; the outcome depends on the specific properties of the niche it creates. Increasing the concentration of an engineered hydrogel from 10% to 16%, corresponding to an approximately fivefold increase in stiffness, significantly improved the survival of transplanted NSCs in the injured spinal cord. In vitro, stiffer substrates enhanced NSC adhesion, spreading, and viability. Pharmacological inhibition or siRNA-mediated suppression of Piezo1 reduced adhesion predominantly on the stiffer substrates, whereas CRISPR/Cas9-mediated disruption of Piezo1 decreased the survival of NSCs transplanted with the 16% hydrogel in vivo. These findings demonstrate that the mechanical properties of the transplantation niche can directly influence graft survival through Piezo1-dependent mechanotransduction [144].

Molecular functionalization of the material provides another level of control. An IGF-1-bioactive supramolecular nanofiber hydrogel activated downstream IGF-1 signaling, reduced NSC apoptosis, enhanced proliferation, and promoted differentiation toward neuronal and oligodendroglial lineages. Following transplantation of NSCs with the IGF-1 hydrogel into the SCI lesion, enhanced neurite outgrowth and myelin sheath regeneration were observed. The material also altered the cargo of extracellular vesicles (EVs) secreted by NSCs and their differentiated progeny, enriching them in miRNAs associated with axonal regeneration and remyelination. These EVs were taken up by endogenous NSCs and altered their differentiation, demonstrating that a biomaterial can influence not only transplanted cells themselves but also their paracrine communication with host cells [101].

Biomaterial-assisted cell transplantation can therefore be viewed as the formation of a local regenerative niche whose properties influence not only graft retention and survival but also its differentiation and interactions with host tissue cells. Graft survival is necessary but insufficient as a criterion of therapeutic efficacy. Evaluation of the resulting interface should also consider graft cell fate, process outgrowth, remyelination, paracrine signaling, and subsequent integration with host tissue.

### 6.2. 3D Scaffolds for Tissue Bridging and Guided Regeneration

In volumetric neural tissue injuries, a three-dimensional scaffold must support cellular infiltration, axonal and vascular ingrowth, and integration with host tissue. Its architecture should therefore provide the spatial organization required to guide regeneration across the lesion.

One approach is to create interconnected free space within an injectable material. PEG-based granular hydrogels were composed of microgels containing RGD cell-adhesive and MMP-degradable peptides and were assembled in situ after injection. In a mouse model of SCI, the material alone did not increase astrogliosis or the recruitment of innate immune cells, while co-transplantation with NPCs supported graft survival, neuronal and astrocytic differentiation, and extension of axonal projections from the transplanted cells into host tissue. The granular architecture thus supported transplanted cells while providing space for cell migration, matrix remodeling, and integration with the surrounding host tissue [145].

A different strategy involves spatially programming the direction of regeneration. Core–shell hydrogel microfibers were fabricated by coaxial extrusion using oxidized hyaluronic acid, carboxymethyl chitosan, and Matrigel, with a high density of NSCs encapsulated within the inner compartment [146]. In vitro, the encapsulated NSCs differentiated into neurons and astrocytes more efficiently than cells cultured on a conventional surface. For transplantation, the microfibers were further reinforced with PLA nanosheets. In a complete spinal cord transection model, the construct filled the lesion and was associated with increased numbers of neuron- and oligodendrocyte-like cells at the lesion epicenter, while regenerating axons were observed across the entire lesion along the microfibers. These findings demonstrate the functional importance of spatially organized constructs as guidance scaffolds for axonal bridging across a large tissue defect.

However, restoration of neural connectivity requires reconstruction not only of the neuronal component but also of the vascular and immune components of the injured tissue. A multicomponent system based on a hyaluronic acid hydrogel and decellularized brain matrix incorporated human embryonic stem cell-derived NPCs, PDRN, and extracellular vesicles derived from TNF-α/IFN-γ-primed mesenchymal stem cells. In a spinal cord transection model, the combined scaffold simultaneously enhanced measures of angiogenesis and neural differentiation while reducing inflammatory and apoptotic responses [147]. Repair of a volumetric defect may therefore require coordination of multiple processes within a single construct, as the maintenance and integration of regenerating neural tissue depend on the establishment of a supportive vascular and cellular microenvironment.

These studies position the three-dimensional regenerative scaffold as a spatial bridge between the margins of injured tissue. Its effectiveness depends on its ability to simultaneously provide space for cellular infiltration and remodeling, guide axonal growth, and support the establishment of a tissue microenvironment within the defect. The relevant criterion for architectural design is therefore not any single structural parameter of the material, but the ability of the construct to support the transition from defect filling to the formation of an integrated tissue bridge.

### 6.3. Modulation of the Neuron–Glia–ECM Microenvironment During Regeneration

The regenerative interface interacts not only with transplanted cells but also with glial populations at the injury site. Its effectiveness therefore depends on whether the resulting microenvironment supports coordinated neuronal and glial responses. This is particularly important in SCI, where microglia and astrocytes reciprocally regulate inflammation, ECM organization, and the accessibility of the lesion to regenerating axons.

Astrocyte-derived ECM illustrates how matrix composition can influence several components of this response. For example, decellularized ECM derived from mESC-derived protoplasmic or fibrous astrocytes was incorporated into hyaluronic acid hydrogels. Both ECM types promoted motoneuron neurite extension at specific concentrations, but protoplasmic astrocyte-derived ECM was effective at a lower ECM content and supported greater neurite outgrowth than fibrous astrocyte-derived ECM at the same ECM-to-HA ratio. Following implantation after SCI in rats, hydrogels containing protoplasmic astrocyte-derived ECM reduced the GFAP-associated glial scar area and macrophage/microglia staining while increasing axonal penetration into the lesion; comparable effects were not observed with fibrous astrocyte-derived ECM [148]. The response to an ECM-containing material therefore depends not simply on the presence of matrix components but also on their cellular source, which can influence neuronal and glial responses simultaneously.

A more targeted strategy is to disrupt pathological feedback between glial cells and the ECM. A recent SCI study identified CSPGs as regulators of a self-reinforcing microglia–astrocyte axis in which CSPG accumulation promoted a pro-inflammatory microglial state and subsequent development of scar-forming astrocytes. To disrupt this pathological feedback loop, a ROS-responsive fusogenic lipopolyplex decorated with the connective tissue growth factor-binding peptide DAG was used to deliver the chondroitinase ABC gene to reactive astrocytes. CSPG degradation altered both microglial and astrocytic responses, reduced glial scarring, and improved structural and functional measures of recovery [149]. Here, the therapeutic target is not an isolated cell population but an interconnected microglia–astrocyte–ECM system that sustains the adverse post-injury microenvironment.

The regenerative state of transplanted neural cells can likewise be coupled to modulation of the immune microenvironment. This dual objective was addressed by combining coaxially printed NSC-laden hydrogel microfibers with extracellular vesicles derived from human umbilical cord mesenchymal stem cells. Addition of the EVs attenuated the pro-inflammatory microglial response while enhancing NSC proliferation and neuronal differentiation in culture. After complete spinal cord transection, the combined treatment reduced pro-inflammatory cytokine levels at the lesion site, increased neuronal cell numbers and angiogenesis, and improved recovery compared with NSC-containing microfibers without EVs [150]. This design therefore couples direct support of neural cells with modulation of the surrounding glial environment.

These studies provide a rationale for designing regenerative interfaces around the interdependence of neural cells, glia, and the ECM. Changes in matrix composition, glial state, or intercellular signaling can simultaneously reshape several components of the regenerative microenvironment. Material efficacy therefore cannot be adequately captured by neurite outgrowth or individual markers of glial activation alone.

### 6.4. Dynamic and Microenvironment-Responsive Regenerative Interfaces

The post-injury microenvironment evolves over time, creating an opportunity to coordinate material function with successive stages of injury and repair. This temporal coordination was implemented in a ROS- and MMP-responsive hydrogel designed for sequential release of therapeutic components. Elevated ROS levels triggered an early antioxidant and anti-inflammatory response, whereas subsequent MMP-dependent release of VEGF promoted angiogenesis and neural stem cell differentiation. Across models of severe SCI, hydrogel treatment reduced inflammation, fibrosis, and lesion cavity formation while enhancing vascularization and neural regeneration and improving functional recovery [151].

The timing of therapeutic release is also relevant to modulation of the immune response. In a hyaluronic acid-based hydrogel, sequential delivery of minocycline and IL-4 was designed to suppress the early pro-inflammatory microglia/macrophage response and subsequently support an anti-inflammatory state, respectively. Compared with simultaneous delivery, the sequential regimen more effectively reduced inflammation and glial scar formation and was accompanied by enhanced neuronal regeneration and locomotor recovery [152].

An alternative strategy is to couple therapeutic activity directly to pathological signals within the injury microenvironment. In a ROS-responsive system based on a zinc-citrate metal–organic framework, elevated ROS triggered the release of H_2_S. In SCI, this system attenuated oxidative stress and inflammation, altered the macrophage state, and supported neuroprotection and angiogenesis [153]. Dynamic material design can therefore operate through two complementary principles: programmed intervention at successive stages of regeneration and adaptive activation in response to the local pathological microenvironment.

## 7. Chronic Neural Interfaces

### 7.1. Mechanical and Structural Integration with Neural Tissue

In chronic neural interfaces, mechanical compatibility influences not only the tissue response but also the stability of functional contact between the active regions of the device and neural tissue. Unlike regenerative materials, an implanted neural interface remains within the tissue over extended periods, and relative micromotion between the device and surrounding tissue arising from respiration, vascular pulsation, and movement can generate repetitive deformation at the interface. For rigid intracortical probes, such micromotion is considered one of the factors contributing to chronic blood–brain barrier (BBB) disruption, microglial and astrocytic activation, and neuronal loss in the vicinity of the implant [23,154]. The resulting mechanical interaction is determined not only by the material properties discussed above but also by device geometry, thickness, fixation, and the dimensions of the region in direct contact with the tissue.

This principle is exemplified by e-dura, a soft epidural neural interface integrating stretchable interconnects, electrodes, and microfluidic chemotrodes [26]. The device was designed to match the shape and deformability of the dura mater and retained functionality through millions of mechanical stretch cycles, electrical stimulation pulses, and chemical injections. Following epidural implantation in rats, e-dura established a mechanically compliant interface with the spinal cord, whereas a stiffer implant produced marked tissue deformation and neuroinflammation. The device also enabled the extraction of cortical states and the delivery of electrochemical spinal neuromodulation. In this system, long-term integration was achieved not by minimizing a single mechanical parameter, but by matching the deformability of the device as a whole to the dynamics of the surrounding tissue.

For intracortical interfaces, another strategy is to reduce both bending stiffness and the cross-sectional dimensions of the implant. Nanoelectronic thread (NET) probes with subcellular dimensions and ultraflexible mechanics maintained stable impedance, noise levels, single-unit recording yield, and signal amplitude during chronic implantation, with recordings obtained for four months. Longitudinal in vivo two-photon imaging and subsequent histological analysis revealed recovery of the local vasculature and BBB, together with an absence of chronic neuronal loss and glial scar formation around the probes [155]. Reducing the mechanical and spatial footprint of an intracortical device can therefore limit chronic disruption of the local cellular and vascular microenvironment while preserving electrical access to neurons.

The same reduction in bending stiffness that improves mechanical compatibility after implantation, however, also makes insertion more difficult because an ultraflexible probe can buckle or deform before entering the tissue. Temporary insertion shuttles, dissolvable or detachable supports, and related delivery strategies are therefore used to separate the mechanical requirements of implantation from those of the chronic interface [154]. Such approaches allow a device to be mechanically supported during insertion while retaining high flexibility after deployment. Mechanical integration of a chronic neural interface is consequently a problem of optimizing the device as a whole rather than simply selecting a material with a low Young’s modulus. Current strategies combine reductions in bending stiffness, device dimensions, and mechanical loading of the tissue with sufficient structural stability for implantation and stable positioning of active sites relative to neural tissue.

### 7.2. Controlling the Biological Boundary—From Antifouling to Selective Biointegration

In chronic neural interfaces, surface interactions influence not only the foreign body response but also the maintenance of functional proximity between neurons and the neural interface. The formation of protein and cellular layers, microglial and astrocytic responses, and remodeling of the local ECM can increase the spatial and functional separation between electrodes and neurons [23]. Surface engineering should therefore aim not to eliminate tissue interactions altogether, but to limit nonspecific biofouling while preserving favorable neuronal contact.

The feasibility of this approach was demonstrated with an ultrathin polymer coating deposited on flexible multifunctional neural probes by photoinitiated chemical vapor deposition. The coating markedly reduced albumin and fibrinogen adsorption without increasing electrical impedance. During chronic implantation in the mouse brain, coated probes maintained high-quality electrophysiological recordings for 13 weeks and exhibited a 66.6% reduction in glial scarring and an 84.6% increase in neuronal preservation compared with uncoated probes [156]. Suppressing nonspecific interfacial interactions can therefore reduce the glial response while preserving the functional performance of a neural interface.

An alternative approach is to selectively enhance neuronal interactions with the device surface. The neuronal cell adhesion molecule L1 was covalently immobilized on intracortical silicon electrode arrays. Over 16 weeks, L1-coated probes exhibited higher visually evoked single-unit yield, single-unit signal amplitude, and signal-to-noise ratio than uncoated arrays. Histological analysis at 16 weeks further revealed greater neuronal and axonal density together with reduced microglial activation around L1-coated probes [157]. Rather than broadly suppressing cell adhesion, the surface of a neural interface can therefore be functionalized to favor neuronal contact while limiting undesirable glial interactions.

Together, these approaches suggest that the optimal surface of a chronic neural interface does not lie at either extreme of the adhesion–antifouling continuum. Antifouling coatings limit nonspecific protein adsorption and glial encapsulation, whereas neuroadhesive functionalization can help maintain neurons in close functional proximity to the electrode. A more relevant design objective is selective biointegration, in which undesirable protein and glial interactions are minimized without compromising favorable interactions with the target neuronal population.

### 7.3. From Bioinert Electrodes to Biomimetic Electronic Tissues

The next stage in the development of chronic neural interfaces extends beyond minimizing material–tissue interactions toward interfaces that reproduce selected properties of the ECM. Within this concept, the electronic component retains its recording or stimulation functions, while the tissue-facing interface becomes highly hydrated, mechanically compliant, and capable of interacting with bioactive molecules. In the electronic tissue concept proposed by Minev, conductive polymer networks are integrated within hydrated matrices, combining electronic and ionic charge transport with tissue-like mechanical properties [158]. Rather than focusing primarily on reducing mechanical mismatch, such materials could potentially reproduce selected ECM functions, including the binding, local sequestration, and controlled release of signaling molecules.

An ECM-like matrix can be integrated directly with electrode surfaces by assembling PEG–heparin hydrogels on ITO, gold, and PtIr electrodes through electrochemically controlled click chemistry [159]. The method enabled spatially localized hydrogel coatings and patterns with different cell-adhesive properties. A hydrated, bioactive layer can therefore be formed directly on an electronic surface, creating an intermediate environment between the electrode and surrounding cells.

Extending this concept to an electronically conductive matrix, a PEDOT:sGAG hydrogel combines a conducting PEDOT network with sulfated glycosaminoglycans. At a PEDOT content of approximately 1 wt.%, the material retained high water content and tissue-like softness, while the sGAG component provided affinity for bioactive proteins, including growth factors. Low-voltage stimulation modulated their retention and release, allowing an electrical input to alter the local availability of biochemical signals [160]. The material therefore functions not only as an electrical conductor but also as an electrically addressable matrix capable of regulating molecular cues.

These systems broaden the functional role of a neural interface from that of an electrode toward an active boundary between electronics and the cellular microenvironment. This principle is particularly relevant to the regulation of neuron–glia crosstalk because it could eventually enable spatial and temporal control over the availability of signals to different cell populations. Direct evidence that such materials can regulate neuron–glia interactions over extended periods in vivo, however, is still lacking. Electronic tissues should therefore be regarded as a promising but still emerging direction in biointegrative neural interfaces.

## 8. Critical Gaps and Unresolved Questions

Despite the substantial body of evidence on interactions between neural tissue and biomaterials, the transferability and causal interpretation of findings across experimental systems remain limited. These limitations arise from differences between 2D and 3D in vitro models and in vivo systems, insufficient observation periods, interdependence among material properties, and the difficulty of distinguishing direct material effects from secondary multicellular responses.

### 8.1. Model Mismatch Across 2D, 3D, and In Vivo Systems

Relationships identified in 2D systems cannot be directly extrapolated to 3D or in vivo models because each captures different components of the material–tissue interface. Two-dimensional systems allow individual parameters such as stiffness, ligand density, or surface chemistry to be examined under comparatively controlled conditions and are therefore particularly useful for dissecting cellular mechanisms. However, cells interact with the substrate in a geometry fundamentally different from that of a three-dimensional matrix, where mechanical and biochemical cues are experienced in the context of spatial confinement, matrix remodeling, and cell–cell interactions. Three-dimensional models reproduce selected features of the ECM and multicellular organization more closely, but this increased complexity also makes experimental variables more difficult to control and cellular responses more difficult to resolve [161].

Moving to in vivo models introduces additional components that remain difficult to reproduce fully even in sophisticated 3D systems, including the vasculature, systemic and local immune responses, the BBB, mechanical loading, and dynamic tissue remodeling. Increasing model complexity should therefore not be viewed simply as a linear progression toward a more faithful representation of the native response. In vitro, organoid, ex vivo, xenograft, and in vivo systems retain distinct strengths and limitations and address different experimental questions [162].

This mismatch becomes particularly apparent when biomaterial performance is compared across experimental settings. A comparison of five 3D-printable biomaterials across physicomechanical, cell–material interaction, and early in vivo biocompatibility assessments illustrates this discrepancy. GelMA exhibited the highest cell viability index at 7 DIV, yet after implantation all materials became surrounded by connective tissue capsules accompanied by inflammatory cells, with the magnitude of the response differing among materials [163]. In vitro cytocompatibility therefore does not directly predict the tissue response in vivo.

### 8.2. Temporal Mismatch and Insufficient Chronic Observation

A second limitation arises from the mismatch between experimental duration and the dynamics of the material–tissue interface. Protein adsorption occurs within minutes to hours, early cellular responses develop over days, whereas tissue remodeling, material degradation, and chronic responses to implanted devices can continue for weeks, months, and potentially years. Measurements obtained at a single time point therefore capture only one phase of the evolving interaction between the material and tissue.

For regenerative systems, a favorable early cellular response does not necessarily predict successful long-term integration. A material may initially support cell survival and adhesion but subsequently restrict migration, axonal ingrowth, or tissue remodeling if its structure persists longer than required. Conversely, overly rapid degradation may cause the material to lose its guidance or mechanical function before new tissue has formed. Measurements of cell survival, inflammatory markers, or residual material volume therefore characterize particular stages of regeneration and should not be interpreted in isolation as measures of overall material efficacy.

For chronic neural interfaces, observation periods are often short relative to the intended operational lifetime of the device. Early reductions in protein adsorption, GFAP, or Iba1 do not guarantee persistence of these effects because the state of the surrounding tissue, coating integrity, electrochemical properties, and mechanical interactions between the device and tissue can all change over time. Moreover, the foreign body response evolves through overlapping inflammatory, glial, and tissue-remodeling processes, making short-term observations insufficient for extrapolation to the chronic phase [164]. This distinction is particularly important for implantable neural interfaces, where functional performance depends not on minimizing the tissue response at an isolated time point but on maintaining stable recording or stimulation throughout the implantation period.

### 8.3. Cellular Scope and Tissue- and Model-Specific Responses

This review focuses primarily on neurons, astrocytes, and microglia, reflecting the cellular populations most directly addressed in the studies of biomaterial-mediated neuron–glia interactions discussed here. Neural tissue responses to injury and biomaterial implantation, however, involve additional cell populations that may influence regenerative outcomes, inflammation, and the long-term integration of implanted materials.

Oligodendrocytes and oligodendrocyte precursor cells (OPCs) are important for myelination and remyelination. In regenerative biomaterial studies, an IGF-1-functionalized hydrogel influenced neural stem cell differentiation toward neuronal and oligodendroglial lineages and modified the biological activity of extracellular vesicles released by these cells [101]. In another study, transplantation of neural stem cells within hydrogel microfibers was associated with an increased number of oligodendrocyte-like cells at the spinal cord lesion site [146]. These findings suggest the involvement of oligodendroglial populations in biomaterial-associated regenerative responses, although the identification of oligodendrocyte-like cells or the expression of lineage markers does not, by itself, establish functional remyelination. Oligodendroglial cells are also mechanosensitive: increased substrate stiffness inhibited oligodendrocyte branching and differentiation [165], while age-associated stiffening of the CNS niche impaired OPC proliferation and differentiation through Piezo1-dependent mechanotransduction [166]. These observations suggest that biomaterial properties may influence oligodendroglial responses independently of, or alongside, astrocyte- and microglia-mediated effects.

Infiltrating monocyte-derived macrophages represent another important component of the tissue response, particularly when injury or implantation compromises the BBB or blood–spinal cord barrier. These cells cannot be reliably distinguished from resident microglia using Iba1 or CD11b immunoreactivity alone. TMEM119 has been proposed as a marker that discriminates microglia from other macrophage populations, although marker-based identification should be interpreted in the context of experimental models [167]. Following spinal cord injury, resident microglia and infiltrating macrophages show distinct phagocytic responses [168], and a population of infiltrating blood-derived macrophages contributes to inflammation resolution and recovery [169]. Macrophage-associated MMP-9 activity has also been implicated in fibronectin-rich ECM remodeling following implantation of an injectable hydrogel into injured spinal cord tissue [42]. These findings underscore that inflammatory and regenerative responses observed in biomaterial studies cannot always be attributed exclusively to resident microglia.

Vascular-associated cells, including pericytes and perivascular fibroblasts, also contribute to tissue remodeling following CNS injury. Experimental studies have identified perivascular cell populations involved in fibrotic scar formation after spinal cord injury [170,171]. In particular, reducing scar formation by a specific pericyte population promoted axonal regeneration and functional recovery in a mouse model of spinal cord injury [172]. The involvement of non-neural cells is also relevant to biomaterial integration: implantation of degradable ECM hydrogels into ischemic brain lesions has been associated with cellular infiltration, material degradation, and formation of new tissue within the lesion cavity [43]. Oligodendroglial, infiltrating immune, and vascular-associated populations can therefore influence outcomes commonly evaluated in biomaterial studies, although their interactions with neurons, astrocytes, and microglia are beyond the scope of this review.

The interpretation of biomaterial-induced responses is further complicated by differences between neural tissues and experimental injury models. The brain and spinal cord differ in their anatomical organization and mechanical environment, and the response to a material may also depend on the pathological state of the surrounding tissue. For example, studies comparing hydrogel implantation in intact brain tissue and ischemic lesions have demonstrated that the local tissue environment influences foreign body responses and biomaterial performance [34]. Spinal cord injury models introduce additional variability. Contusion, hemisection, and complete transection differ in lesion geometry, the extent of spared tissue, and the continuity of neural pathways, creating distinct requirements for scaffold integration and regeneration [42,136,137]. Consequently, regenerative effects observed in one injury model cannot be assumed to occur under different anatomical or pathological conditions.

Similar considerations apply to chronic neural interfaces and neuronal culture models. Many studies of electrode–tissue interactions have focused on intracortical implantation, whereas spinal interfaces operate under different anatomical and mechanical conditions [23,26]. Neuronal responses to mechanical cues may also differ between CNS and PNS populations. For example, substrate stiffness affected neurite growth differently in hippocampal neurons and DRG neurons [44]. Experimental modulation of Piezo1 signaling influenced stiffness-dependent axonal growth in DRG neurons and regeneration following peripheral nerve injury [87], but these findings do not establish equivalent effects in injured CNS axons. The biological effects of a biomaterial should therefore be interpreted in relation to the specific cellular composition, neural tissue, injury model, and implantation conditions rather than generalized across experimental systems.

### 8.4. Methodological Approaches to Resolving Current Limitations

Addressing these limitations requires experimental approaches capable of distinguishing direct material-induced cellular responses from secondary effects mediated by intercellular communication. Compartmentalized microfluidic co-culture systems provide spatial control over neuronal and glial populations and allow localized experimental manipulation. Such platforms have been used to isolate neuronal axons from cell bodies and to investigate interactions between CNS axons and oligodendrocytes [173,174]. Their adaptation to biomaterial-based co-culture models could help distinguish direct material effects from indirect responses mediated by neuron–glia interactions.

Cell-type-specific optogenetic stimulation offers a complementary approach for investigating the functional consequences of intercellular signaling. Selective optogenetic activation of astrocytes has been shown to modulate neuronal synaptic transmission and activity in vivo [175]. Combined with calcium imaging, electrophysiological recordings, or measurements of extracellular mediators, similar approaches could help determine whether material-induced changes in the activity of one cell population contribute to functional responses in neighboring cells.

Single-cell transcriptomic approaches provide another means of resolving cellular heterogeneity. Single-cell RNA sequencing has revealed molecularly distinct neuronal and non-neuronal populations in the mammalian brain [176], while integrative computational approaches enable the combined analysis of transcriptomic and chromatin accessibility data [177]. Applied to biomaterial-based neural models, these methods could help identify cell-type-specific molecular responses and regulatory changes that remain obscured in bulk analyses. However, molecular profiles alone cannot establish functional intercellular communication and should therefore be interpreted alongside spatially resolved measurements and functional assays.

Together, these complementary approaches may improve the mechanistic interpretation of biomaterial-induced cellular responses by combining spatial control, selective perturbation, and cell-type-resolved molecular characterization. Their integration with longitudinal measurements and appropriately controlled material comparisons could help establish more direct links between material properties, neuron–glia communication, and functional outcomes.

## 9. Conclusions

Biomaterials research is progressively moving beyond assessments of cytocompatibility toward understanding how materials influence the organization and function of neural tissue. Yet neuronal, astrocytic, and microglial responses are still frequently examined in isolation. Although this approach can establish how a material affects neuronal viability, neurite growth, or glial reactivity, it does not adequately capture the interactions among cell populations that contribute to neural tissue regeneration and long-term integration of neural interfaces.

The studies discussed in this review also show that biomaterial effects cannot be attributed to a single isolated property. Mechanics, architecture, surface chemistry, electrical conductivity, and degradation collectively shape cell–material interactions and may differentially affect the neuronal and glial components of neural tissue. Optimal material properties therefore depend on the intended function. In regenerative systems, materials should provide temporary cellular support, guide neural process growth, and permit subsequent tissue remodeling. Chronic neural interfaces, by contrast, require sustained functional proximity to neurons while limiting undesirable protein adsorption and glial responses.

Importantly, concurrent improvement in neuronal outcomes and attenuation of astrocytic or microglial reactivity does not by itself demonstrate that a material directly modulates neuron–glia crosstalk. Establishing such a relationship requires studies that combine controlled variation in material properties with analysis of intercellular signaling and experimental perturbation of the proposed mechanisms of cell–cell interaction. Models incorporating multiple cell populations and enabling their states and interactions to be followed over time will be particularly important for this purpose.

The next step in the development of biomaterials for neuroscience is therefore to move beyond the independent optimization of individual cellular responses toward control of interactions among neurons, glia, and the ECM. This framework should make it possible to determine more precisely which material properties contribute to neural tissue regeneration or the long-term performance of neural interfaces and to establish more mechanistically grounded principles for their design.

## Data Availability

No new data were created or analyzed in this study. Data sharing is not applicable to this article.

## Abbreviations

The following abbreviations are used in this manuscript:

ASC	Apoptosis-associated speck-like protein containing a CARD
ATP	Adenosine triphosphate
BBB	Blood–brain barrier
CaMKII	Ca^2+^/calmodulin-dependent protein kinase II
CNS	Central nervous system
CNT	Carbon nanotube
CSPG	Chondroitin sulfate proteoglycan
DAMPs	Damage-associated molecular patterns
DCM	Dichloromethane
DIV	Days in vitro
DRG	Dorsal root ganglion
ECM	Extracellular matrix
ERK	Extracellular signal-regulated kinase
EVs	Extracellular vesicles
FAK	Focal adhesion kinase
GABA	Gamma-aminobutyric acid
GelMA	Gelatin methacryloyl
GFAP	Glial fibrillary acidic protein
GO	Graphene oxide
HA	Hyaluronic acid
HFIP	1,1,1,3,3,3-Hexafluoro-2-propanol
IFN	Interferon
IGF	Insulin-like growth factor
IgG	Immunoglobulin G
IKVAV	Isoleucine-lysine-valine-alanine-valine
IL	Interleukin
ITO	Indium tin oxide
JAK	Janus kinase
L1	Neuronal cell adhesion molecule L1
LBA	Lactobionic acid
LCS	Lithium–calcium–silicate
LPS	Lipopolysaccharide
MAP2	Microtubule-associated protein 2
MEK	Mitogen-activated protein kinase kinase
MMP	Matrix metalloproteinase
NET	Nanoelectronic thread
NF-κB	Nuclear factor kappa B
NPC	Neural progenitor cell
NSC	Neural stem cell
NSPC	Neural stem/progenitor cell
PCL	Poly(ε-caprolactone)
PDMS	Polydimethylsiloxane
PDRN	Polydeoxyribonucleotide
PEDOT	Poly(3,4-ethylenedioxythiophene)
PEG	Poly(ethylene glycol)
PGA	Poly(glycolic acid)
PI3K	Phosphoinositide 3-kinase
PLA	Poly(lactic acid)
PLGA	Poly(lactic-co-glycolic acid)
PLL	Poly(L-lysine)
PLLA	Poly(L-lactic acid)
PSBMA	Poly(sulfobetaine methacrylate)
PSS	Poly(styrene sulfonate)
RGD	Arginine-glycine-aspartate
RhoA	Ras homolog family member A
ROCK	Rho-associated coiled-coil kinase
ROS	Reactive oxygen species
SCI	Spinal cord injury
sGAG	Sulfated glycosaminoglycan
siRNA	Small interfering RNA
STAT3	Signal transducer and activator of transcription 3
TAZ	Transcriptional coactivator with PDZ-binding motif
TCF/LEF	T-cell factor/lymphoid enhancer factor
TEAD	TEA domain transcription factor
THF	Tetrahydrofuran
TNF	Tumor necrosis factor
VEGF	Vascular endothelial growth factor
YAP	Yes-associated protein

## References

[1] Steinmetz J.D., Seeher K.M., Schiess N., Nichols E., Cao B., Servili C., Cavallera V., Cousin E., Hagins H., Moberg M.E. (2024). Global, Regional, and National Burden of Disorders Affecting the Nervous System, 1990–2021: A Systematic Analysis for the Global Burden of Disease Study 2021. Lancet Neurol..

[2] Maas A.I.R., Menon D.K., Adelson P.D., Andelic N., Bell M.J., Belli A., Bragge P., Brazinova A., Büki A., Chesnut R.M. (2017). Traumatic Brain Injury: Integrated Approaches to Improve Prevention, Clinical Care, and Research. Lancet Neurol..

[3] Safdarian M., Trinka E., Rahimi-Movaghar V., Thomschewski A., Aali A., Abady G.G., Abate S.M., Abd-Allah F., Abedi A., Adane D.E. (2023). Global, Regional, and National Burden of Spinal Cord Injury, 1990–2019: A Systematic Analysis for the Global Burden of Disease Study 2019. Lancet Neurol..

[4] Diop M., Epstein D. (2024). A Systematic Review of the Impact of Spinal Cord Injury on Costs and Health-Related Quality of Life. PharmacoEcon. Open.

[5] Pai V., Singh B.N., Singh A.K. (2024). Insights into Advances and Applications of Biomaterials for Nerve Tissue Injuries and Neurodegenerative Disorders. Macromol. Biosci..

[6] Boufidis D., Garg R., Angelopoulos E., Cullen D.K., Vitale F. (2025). Bio-Inspired Electronics: Soft, Biohybrid, and “Living” Neural Interfaces. Nat. Commun..

[7] Niyomsri S., Duarte R.V., Eldabe S., Fiore G., Kopell B.H., McNicol E., Taylor R.S. (2020). A Systematic Review of Economic Evaluations Reporting the Cost-Effectiveness of Spinal Cord Stimulation. Value Health.

[8] Ghuman H., Modo M. (2016). Biomaterial Applications in Neural Therapy and Repair. Chin. Neurosurg. J..

[9] Jayakumar Choudhury P.W., Navya V.B., Jena S., Somu P., Rajkumar S., Das M., Kumar R. (2024). Biological Response to Biomaterials. Smart Ways of Biomaterial Designing Synthesis and Characterization.

[10] Schmidt D.R., Waldeck H., Kao W.J., Puleo D.A., Bizios R. (2009). Protein Adsorption to Biomaterials. Biological Interactions on Materials Surfaces.

[11] Monopoli M.P., Åberg C., Salvati A., Dawson K.A. (2012). Biomolecular Coronas Provide the Biological Identity of Nanosized Materials. Nat. Nanotech.

[12] Gräfe C., Von Der Lühe M., Weidner A., Globig P., Clement J.H., Dutz S., Schacher F.H. (2019). Protein Corona Formation and Its Constitutional Changes on Magnetic Nanoparticles in Serum Featuring a Polydehydroalanine Coating: Effects of Charge and Incubation Conditions. Nanotechnology.

[13] Kasemo B., Lausmaa J. (1994). Material-Tissue Interfaces: The Role of Surface Properties and Processes. Environ. Health Perspect..

[14] Meng Q., Zhang M., Zhang Y., Jiang H., Qu Y., Li C., Hu C., Lin Z., Xing B., Zhu F. (2025). Advances in Adhesion Interfaces of Peripheral Nerve Repair Materials: Mechanism, Classification, Evaluation Method, and Functionalization. Front. Cell Dev. Biol..

[15] Zuidema J.M., Gilbert R.J., Gottipati M.K. (2018). Biomaterial Approaches to Modulate Reactive Astroglial Response. Cells Tissues Organs.

[16] Liu L., Liu J., Bao J., Bai Q., Wang G. (2020). Interaction of Microglia and Astrocytes in the Neurovascular Unit. Front. Immunol..

[17] Goldberg J.L. (2012). Role of Electrical Activity in Promoting Neural Repair. Neurosci. Lett..

[18] Liddelow S.A., Barres B.A. (2017). Reactive Astrocytes: Production, Function, and Therapeutic Potential. Immunity.

[19] Faust T.E., Lee Y.-H., O’Connor C.D., Boyle M.A., Gunner G., Durán-Laforet V., Ferrari L.L., Murphy R.E., Badimon A., Sakers K. (2025). Microglia-Astrocyte Crosstalk Regulates Synapse Remodeling via Wnt Signaling. Cell.

[20] Liddelow S.A., Guttenplan K.A., Clarke L.E., Bennett F.C., Bohlen C.J., Schirmer L., Bennett M.L., Münch A.E., Chung W.-S., Peterson T.C. (2017). Neurotoxic Reactive Astrocytes Are Induced by Activated Microglia. Nature.

[21] Frosch M., Shimizu T., Wogram E., Amann L., Gruber L., Groisman A.I., Fliegauf M., Schwabenland M., Chhatbar C., Zechel S. (2025). Microglia–Neuron Crosstalk through Hex–GM2–MGL2 Maintains Brain Homeostasis. Nature.

[22] Sands I., Demarco R., Thurber L., Esteban-Linares A., Song D., Meng E., Chen Y. (2024). Interface-Mediated Neurogenic Signaling: The Impact of Surface Geometry and Chemistry on Neural Cell Behavior for Regenerative and Brain–Machine Interfacing Applications. Adv. Mater..

[23] Salatino J.W., Ludwig K.A., Kozai T.D.Y., Purcell E.K. (2017). Glial Responses to Implanted Electrodes in the Brain. Nat. Biomed. Eng..

[24] Khavandegar A., Safari Dehnavi N., Ganau M., Ramezani Z., Khodadoust E., Hassan Zadeh Tabatabaei M.S., Maleki T., Mousavi M.A., RayatSanati K., Esmaeili M. (2025). Neuroregenerative and Neuroprotective Effects of Bioengineered Scaffolds in Spinal Cord Injury: A Systematic Review of Preclinical and Early Phase Clinical Studies. Spinal Cord.

[25] Bradshaw K.J., Leipzig N.D. (2025). Applications of Regenerative Tissue-Engineered Scaffolds for Treatment of Spinal Cord Injury. Tissue Eng. Part A.

[26] Minev I.R., Musienko P., Hirsch A., Barraud Q., Wenger N., Moraud E.M., Gandar J., Capogrosso M., Milekovic T., Asboth L. (2015). Electronic Dura Mater for Long-Term Multimodal Neural Interfaces. Science.

[27] Cedervall T., Lynch I., Lindman S., Berggård T., Thulin E., Nilsson H., Dawson K.A., Linse S. (2007). Understanding the Nanoparticle–Protein Corona Using Methods to Quantify Exchange Rates and Affinities of Proteins for Nanoparticles. Proc. Natl. Acad. Sci. USA.

[28] Tengvall P. (2011). Protein Interactions with Biomaterials. Comprehensive Biomaterials.

[29] Vilaseca P., Dawson K.A., Franzese G. (2013). Understanding and Modulating the Competitive Surface-Adsorption of Proteins through Coarse-Grained Molecular Dynamics Simulations. Soft Matter.

[30] Noh H., Vogler E.A. (2007). Volumetric Interpretation of Protein Adsorption: Competition from Mixtures and the Vroman Effect. Biomaterials.

[31] Richter-Bisson Z.W., Hedberg Y.S. (2025). Revisiting the Vroman Effect: Mechanisms of Competitive Protein Exchange on Surfaces. Colloids Surf. B Biointerfaces.

[32] Parhi P., Golas A., Barnthip N., Noh H., Vogler E.A. (2009). Volumetric Interpretation of Protein Adsorption: Capacity Scaling with Adsorbate Molecular Weight and Adsorbent Surface Energy. Biomaterials.

[33] Keselowsky B.G., Collard D.M., García A.J. (2003). Surface Chemistry Modulates Fibronectin Conformation and Directs Integrin Binding and Specificity to Control Cell Adhesion. J. Biomed. Mater. Res..

[34] OʼShea T.M., Wollenberg A.L., Kim J.H., Ao Y., Deming T.J., Sofroniew M.V. (2020). Foreign Body Responses in Mouse Central Nervous System Mimic Natural Wound Responses and Alter Biomaterial Functions. Nat. Commun..

[35] Hynes R.O. (2002). Integrins. Cell.

[36] Tucker B.A., Rahimtula M., Mearow K.M. (2005). Integrin Activation and Neurotrophin Signaling Cooperate to Enhance Neurite Outgrowth in Sensory Neurons. J. Comp. Neurol..

[37] Yin Y., Wang W., Shao Q., Li B., Yu D., Zhou X., Parajuli J., Xu H., Qiu T., Yetisen A.K. (2021). Pentapeptide IKVAV-Engineered Hydrogels for Neural Stem Cell Attachment. Biomater. Sci..

[38] Li X., Liu X., Josey B., Chou C.J., Tan Y., Zhang N., Wen X. (2014). Short Laminin Peptide for Improved Neural Stem Cell Growth. Stem Cells Transl. Med..

[39] Zhu L., Plow E.F., Qin J. (2021). Initiation of Focal Adhesion Assembly by Talin and Kindlin: A Dynamic View. Protein Sci..

[40] Madl C.M., LeSavage B.L., Dewi R.E., Dinh C.B., Stowers R.S., Khariton M., Lampe K.J., Nguyen D., Chaudhuri O., Enejder A. (2017). Maintenance of Neural Progenitor Cell Stemness in 3D Hydrogels Requires Matrix Remodelling. Nat. Mater..

[41] Madl C.M., LeSavage B.L., Dewi R.E., Lampe K.J., Heilshorn S.C. (2019). Matrix Remodeling Enhances the Differentiation Capacity of Neural Progenitor Cells in 3D Hydrogels. Adv. Sci..

[42] Hong L.T.A., Kim Y.-M., Park H.H., Hwang D.H., Cui Y., Lee E.M., Yahn S., Lee J.K., Song S.-C., Kim B.G. (2017). An Injectable Hydrogel Enhances Tissue Repair after Spinal Cord Injury by Promoting Extracellular Matrix Remodeling. Nat. Commun..

[43] Ghuman H., Mauney C., Donnelly J., Massensini A.R., Badylak S.F., Modo M. (2018). Biodegradation of ECM Hydrogel Promotes Endogenous Brain Tissue Restoration in a Rat Model of Stroke. Acta Biomater..

[44] Koch D., Rosoff W.J., Jiang J., Geller H.M., Urbach J.S. (2012). Strength in the Periphery: Growth Cone Biomechanics and Substrate Rigidity Response in Peripheral and Central Nervous System Neurons. Biophys. J..

[45] Chi S., Cui Y., Wang H., Jiang J., Zhang T., Sun S., Zhou Z., Zhong Y., Xiao B. (2022). Astrocytic Piezo1-Mediated Mechanotransduction Determines Adult Neurogenesis and Cognitive Functions. Neuron.

[46] Jiang X., Georges P.C., Li B., Du Y., Kutzing M.K., Previtera M.L., Langrana N.A., Firestein B.L. (2007). Cell Growth in Response to Mechanical Stiffness Is Affected by Neuron—Astroglia Interactions. Open Neurosci. J..

[47] Wilson C.L., Hayward S.L., Kidambi S. (2016). Astrogliosis in a Dish: Substrate Stiffness Induces Astrogliosis in Primary Rat Astrocytes. RSC Adv..

[48] Nimmerjahn A., Kirchhoff F., Helmchen F. (2005). Resting Microglial Cells Are Highly Dynamic Surveillants of Brain Parenchyma in Vivo. Science.

[49] Leung B.K., Biran R., Underwood C.J., Tresco P.A. (2008). Characterization of Microglial Attachment and Cytokine Release on Biomaterials of Differing Surface Chemistry. Biomaterials.

[50] Bollmann L., Koser D.E., Shahapure R., Gautier H.O.B., Holzapfel G.A., Scarcelli G., Gather M.C., Ulbricht E., Franze K. (2015). Microglia Mechanics: Immune Activation Alters Traction Forces and Durotaxis. Front. Cell. Neurosci..

[51] Chapman C.A.R., Chen H., Stamou M., Biener J., Biener M.M., Lein P.J., Seker E. (2015). Nanoporous Gold as a Neural Interface Coating: Effects of Topography, Surface Chemistry, and Feature Size. ACS Appl. Mater. Interfaces.

[52] Chapman C.A.R., Chen H., Stamou M., Lein P.J., Seker E. (2016). Mechanisms of Reduced Astrocyte Surface Coverage in Cortical Neuron-Glia Co-Cultures on Nanoporous Gold Surfaces. Cel. Mol. Bioeng..

[53] Lam D., Enright H.A., Cadena J., Peters S.K.G., Sales A.P., Osburn J.J., Soscia D.A., Kulp K.S., Wheeler E.K., Fischer N.O. (2019). Tissue-Specific Extracellular Matrix Accelerates the Formation of Neural Networks and Communities in a Neuron-Glia Co-Culture on a Multi-Electrode Array. Sci. Rep..

[54] Chiacchiaretta M., Bramini M., Rocchi A., Armirotti A., Giordano E., Vázquez E., Bandiera T., Ferroni S., Cesca F., Benfenati F. (2018). Graphene Oxide Upregulates the Homeostatic Functions of Primary Astrocytes and Modulates Astrocyte-to-Neuron Communication. Nano Lett..

[55] O’Connor C., Woods I., McComish S.F., Kerr S., McGrath M., Chávez J.C.P., Maughan J., McGuire T., Caldwell M.A., Dervan A. (2025). Biomimetic Scaffolds Enhance iPSC Astrocyte Progenitor Angiogenic, Immunomodulatory, and Neurotrophic Capacity in a Stiffness and Matrix-Dependent Manner for Spinal Cord Repair Applications. Adv. Healthc. Mater..

[56] Mattotti M., Alvarez Z., Ortega J.A., Planell J.A., Engel E., Alcántara S. (2012). Inducing Functional Radial Glia-like Progenitors from Cortical Astrocyte Cultures Using Micropatterned PMMA. Biomaterials.

[57] Maclean F.L., Lau C.L., Ozergun S., O’Shea R.D., Cederfur C., Wang J., Healy K.E., Walker F.R., Tomas D., Horne M.K. (2017). Galactose-Functionalised PCL Nanofibre Scaffolds to Attenuate Inflammatory Action of Astrocytes in Vitro and in Vivo. J. Mater. Chem. B.

[58] Zhu H., Qiao X., Liu W., Wang C., Zhao Y. (2020). Microglia Play an Essential Role in Synapse Development and Neuron Maturation in Tissue-Engineered Neural Tissues. Front. Neurosci..

[59] Song Q., Jiang Z., Li N., Liu P., Liu L., Tang M., Cheng G. (2014). Anti-Inflammatory Effects of Three-Dimensional Graphene Foams Cultured with Microglial Cells. Biomaterials.

[60] Meng F., Hlady V., Tresco P.A. (2012). Inducing Alignment in Astrocyte Tissue Constructs by Surface Ligands Patterned on Biomaterials. Biomaterials.

[61] Benincasa J.C., Madias M.I., Kandell R.M., Delgado-Garcia L.M., Engler A.J., Kwon E.J., Porcionatto M.A. (2024). Mechanobiological Modulation of In Vitro Astrocyte Reactivity Using Variable Gel Stiffness. ACS Biomater. Sci. Eng..

[62] Ganguly D., Johnson C.D.L., Gottipati M.K., Rende D., Borca-Tasciuc D.-A., Gilbert R.J. (2018). Specific Nanoporous Geometries on Anodized Alumina Surfaces Influence Astrocyte Adhesion and Glial Fibrillary Acidic Protein Immunoreactivity Levels. ACS Biomater. Sci. Eng..

[63] Shtol V.S., Tsareva A.D., Arsentiev K.A., Konovalova S.P., Tlimahova S.A., Klinov D.V., Ivanov D.A., Musienko P.E. (2026). Polylactide/Polycaprolactone Nanofiber Scaffold Enhances Primary Cortical Neuron Growth. Polymers.

[64] Verkhratsky A., Nedergaard M. (2018). Physiology of Astroglia. Physiol. Rev..

[65] Magistretti P.J., Allaman I. (2018). Lactate in the Brain: From Metabolic End-Product to Signalling Molecule. Nat. Rev. Neurosci..

[66] Sofroniew M.V. (2018). Dissecting Spinal Cord Regeneration. Nature.

[67] Escartin C., Galea E., Lakatos A., O’Callaghan J.P., Petzold G.C., Serrano-Pozo A., Steinhäuser C., Volterra A., Carmignoto G., Agarwal A. (2021). Reactive Astrocyte Nomenclature, Definitions, and Future Directions. Nat. Neurosci..

[68] Davalos D., Grutzendler J., Yang G., Kim J.V., Zuo Y., Jung S., Littman D.R., Dustin M.L., Gan W.-B. (2005). ATP Mediates Rapid Microglial Response to Local Brain Injury in Vivo. Nat. Neurosci..

[69] Procès A., Alpizar Y.A., Halliez S., Brône B., Saudou F., Ris L., Gabriele S. (2024). Stretch-Injury Promotes Microglia Activation with Enhanced Phagocytic and Synaptic Stripping Activities. Biomaterials.

[70] Paolicelli R.C., Sierra A., Stevens B., Tremblay M.-E., Aguzzi A., Ajami B., Amit I., Audinat E., Bechmann I., Bennett M. (2022). Microglia States and Nomenclature: A Field at Its Crossroads. Neuron.

[71] Malko P., Jia X., Wood I., Jiang L. (2023). Piezo1 Channel-mediated Ca^2+^ Signaling Inhibits Lipopolysaccharide-induced Activation of the NF-κB Inflammatory Signaling Pathway and Generation of TNF-α and IL-6 in Microglial Cells. Glia.

[72] Herrmann J.E., Imura T., Song B., Qi J., Ao Y., Nguyen T.K., Korsak R.A., Takeda K., Akira S., Sofroniew M.V. (2008). STAT3 Is a Critical Regulator of Astrogliosis and Scar Formation after Spinal Cord Injury. J. Neurosci..

[73] Anderson M.A., Burda J.E., Ren Y., Ao Y., O’Shea T.M., Kawaguchi R., Coppola G., Khakh B.S., Deming T.J., Sofroniew M.V. (2016). Astrocyte Scar Formation Aids Central Nervous System Axon Regeneration. Nature.

[74] Bellver-Landete V., Bretheau F., Mailhot B., Vallières N., Lessard M., Janelle M.-E., Vernoux N., Tremblay M.-È., Fuehrmann T., Shoichet M.S. (2019). Microglia Are an Essential Component of the Neuroprotective Scar That Forms after Spinal Cord Injury. Nat. Commun..

[75] Micholt L., Gärtner A., Prodanov D., Braeken D., Dotti C.G., Bartic C. (2013). Substrate Topography Determines Neuronal Polarization and Growth In Vitro. PLoS ONE.

[76] Robles E., Gomez T.M. (2006). Focal Adhesion Kinase Signaling at Sites of Integrin-Mediated Adhesion Controls Axon Pathfinding. Nat. Neurosci..

[77] Koser D.E., Thompson A.J., Foster S.K., Dwivedy A., Pillai E.K., Sheridan G.K., Svoboda H., Viana M., Costa L.D.F., Guck J. (2016). Mechanosensing Is Critical for Axon Growth in the Developing Brain. Nat. Neurosci..

[78] Sun Z., Guo S.S., Fässler R. (2016). Integrin-Mediated Mechanotransduction. J. Cell Biol..

[79] Mitra S.K., Hanson D.A., Schlaepfer D.D. (2005). Focal Adhesion Kinase: In Command and Control of Cell Motility. Nat. Rev. Mol. Cell Biol..

[80] Wang D.-Y., Melero C., Albaraky A., Atherton P., Jansen K.A., Dimitracopoulos A., Dajas-Bailador F., Reid A., Franze K., Ballestrem C. (2021). Vinculin Is Required for Neuronal Mechanosensing but Not for Axon Outgrowth. Exp. Cell Res..

[81] Govek E.-E., Newey S.E., Van Aelst L. (2005). The Role of the Rho GTPases in Neuronal Development. Genes Dev..

[82] Hall A., Lalli G. (2010). Rho and Ras GTPases in Axon Growth, Guidance, and Branching. Cold Spring Harb. Perspect. Biol..

[83] Panciera T., Azzolin L., Cordenonsi M., Piccolo S. (2017). Mechanobiology of YAP and TAZ in Physiology and Disease. Nat. Rev. Mol. Cell Biol..

[84] Dupont S., Morsut L., Aragona M., Enzo E., Giulitti S., Cordenonsi M., Zanconato F., Le Digabel J., Forcato M., Bicciato S. (2011). Role of YAP/TAZ in Mechanotransduction. Nature.

[85] Holland E.N., Fernández-Yagüe M.A., Zhou D.W., O’Neill E.B., Woodfolk A.U., Mora-Boza A., Fu J., Schlaepfer D.D., García A.J. (2024). FAK, Vinculin, and Talin Control Mechanosensitive YAP Nuclear Localization. Biomaterials.

[86] Rammensee S., Kang M.S., Georgiou K., Kumar S., Schaffer D.V. (2017). Dynamics of Mechanosensitive Neural Stem Cell Differentiation. Stem Cells.

[87] Lei M., Wang W., Zhang H., Gong J., Cai H., Wang Z., Zhu L., Yang X., Wang S., Ma C. (2024). Piezo1 Regulates Stiffness-Dependent DRG Axon Regeneration via Modifying Cytoskeletal Dynamics. Adv. Sci..

[88] Coste B., Mathur J., Schmidt M., Earley T.J., Ranade S., Petrus M.J., Dubin A.E., Patapoutian A. (2010). Piezo1 and Piezo2 Are Essential Components of Distinct Mechanically Activated Cation Channels. Science.

[89] Murthy S.E., Dubin A.E., Patapoutian A. (2017). Piezos Thrive under Pressure: Mechanically Activated Ion Channels in Health and Disease. Nat. Rev. Mol. Cell Biol..

[90] Pathak M.M., Nourse J.L., Tran T., Hwe J., Arulmoli J., Le D.T.T., Bernardis E., Flanagan L.A., Tombola F. (2014). Stretch-Activated Ion Channel Piezo1 Directs Lineage Choice in Human Neural Stem Cells. Proc. Natl. Acad. Sci. USA.

[91] Dupont S. (2016). Role of YAP/TAZ in Cell-Matrix Adhesion-Mediated Signalling and Mechanotransduction. Exp. Cell Res..

[92] Kang P.H., Schaffer D.V., Kumar S. (2020). Angiomotin Links ROCK and YAP Signaling in Mechanosensitive Differentiation of Neural Stem Cells. MBoC.

[93] Liu Z., Mao S., Hu Y., Liu F., Shao X. (2023). Hydrogel Platform Facilitating Astrocytic Differentiation through Cell Mechanosensing and YAP-Mediated Transcription. Mater. Today Bio.

[94] Zhang H., Qin C., Shi Z., Xue J., Hao J., Huang J., Du L., Lu H., Wu C. (2024). Bioprinting of Inorganic-Biomaterial/Neural-Stem-Cell Constructs for Multiple Tissue Regeneration and Functional Recovery. Natl. Sci. Rev..

[95] Zhao X., Lu X., Li K., Song S., Luo Z., Zheng C., Yang C., Wang X., Wang L., Tang Y. (2023). Double Crosslinked Biomimetic Composite Hydrogels Containing Topographical Cues and WAY-316606 Induce Neural Tissue Regeneration and Functional Recovery after Spinal Cord Injury. Bioact. Mater..

[96] Galindo A.N., Frey Rubio D.A., Hettiaratchi M.H. (2024). Biomaterial Strategies for Regulating the Neuroinflammatory Response. Mater. Adv..

[97] Swanson K.V., Deng M., Ting J.P.-Y. (2019). The NLRP3 Inflammasome: Molecular Activation and Regulation to Therapeutics. Nat. Rev. Immunol..

[98] Franklin M.E., Bennett C., Arboite M., Alvarez-Ciara A., Corrales N., Verdelus J., Dietrich W.D., Keane R.W., De Rivero Vaccari J.P., Prasad A. (2023). Activation of Inflammasomes and Their Effects on Neuroinflammation at the Microelectrode-Tissue Interface in Intracortical Implants. Biomaterials.

[99] Moshayedi P., Ng G., Kwok J.C.F., Yeo G.S.H., Bryant C.E., Fawcett J.W., Franze K., Guck J. (2014). The Relationship between Glial Cell Mechanosensitivity and Foreign Body Reactions in the Central Nervous System. Biomaterials.

[100] Singh A.V., Raymond M., Pace F., Certo A., Zuidema J.M., McKay C.A., Gilbert R.J., Lu X.L., Wan L.Q. (2015). Astrocytes Increase ATP Exocytosis Mediated Calcium Signaling in Response to Microgroove Structures. Sci. Rep..

[101] Song P., Han T., Wu Z., Fang H., Liu Y., Ying W., Wang X., Shen C. (2024). Transplantation of Neural Stem Cells Loaded in an IGF-1 Bioactive Supramolecular Nanofiber Hydrogel for the Effective Treatment of Spinal Cord Injury. Adv. Sci..

[102] Qiao E., Fulmore C.A., Schaffer D.V., Kumar S. (2024). Substrate Stress Relaxation Regulates Neural Stem Cell Fate Commitment. Proc. Natl. Acad. Sci. USA.

[103] Velasco-Estevez M., Rolle S.O., Mampay M., Dev K.K., Sheridan G.K. (2020). Piezo1 Regulates Calcium Oscillations and Cytokine Release from Astrocytes. Glia.

[104] Sáez P., Borau C., Antonovaite N., Franze K. (2023). Brain Tissue Mechanics Is Governed by Microscale Relations of the Tissue Constituents. Biomaterials.

[105] Hou J., Jiang K., Ramanathan A., Kumar A.S., Zhang W., Zhao L., Wu T., Pidaparti R., Zhu D., Li G. (2025). Mechanical Characterization of Brain Tissue: Experimental Techniques, Human Testing Considerations, and Perspectives. Acta Biomater..

[106] Jin C., Yu J., Li R., Ye X. (2024). Regional Biomechanical Characterization of the Spinal Cord Tissue: Dynamic Mechanical Response. Front. Bioeng. Biotechnol..

[107] Neumann O., Surana H.V., Melly S., Steinmann P., Budday S. (2025). Mechanical Characteristics of Spinal Cord Tissue by Indentation. J. Mech. Behav. Biomed. Mater..

[108] Stanners M., O’Riordan M., Theodosiou E., Souppez J.-B.R.G., Gardner A. (2024). The Mechanical Properties of the Spinal Cord: A Systematic Review. Spine J..

[109] Bartlett R.D., Eleftheriadou D., Evans R., Choi D., Phillips J.B. (2020). Mechanical Properties of the Spinal Cord and Brain: Comparison with Clinical-Grade Biomaterials for Tissue Engineering and Regenerative Medicine. Biomaterials.

[110] Small G., Ballatore F., Giverso C., Balbi V. (2025). Modelling the Non-Linear Viscoelastic Behaviour of Brain Tissue in Torsion. Soft Matter.

[111] Saha K., Keung A.J., Irwin E.F., Li Y., Little L., Schaffer D.V., Healy K.E. (2008). Substrate Modulus Directs Neural Stem Cell Behavior. Biophys. J..

[112] Leipzig N.D., Shoichet M.S. (2009). The Effect of Substrate Stiffness on Adult Neural Stem Cell Behavior. Biomaterials.

[113] Kayal C., Moeendarbary E., Shipley R.J., Phillips J.B. (2020). Mechanical Response of Neural Cells to Physiologically Relevant Stiffness Gradients. Adv. Healthc. Mater..

[114] Woods I., O’Connor C., Frugoli L., Kerr S., Gutierrez Gonzalez J., Stasiewicz M., McGuire T., Cavanagh B., Hibbitts A., Dervan A. (2022). Biomimetic Scaffolds for Spinal Cord Applications Exhibit Stiffness-Dependent Immunomodulatory and Neurotrophic Characteristics. Adv. Healthc. Mater..

[115] Brown L.E., Bakker D., Zhou P., Madl C.M. (2026). Stress Relaxation Timescale and Hydrogel Network Connectivity Regulate Neural Progenitor Cell Stemness and Differentiation. J. Mater. Chem. B.

[116] Keith A.N., Vatankhah-Varnosfaderani M., Clair C., Fahimipour F., Dashtimoghadam E., Lallam A., Sztucki M., Ivanov D.A., Liang H., Dobrynin A.V. (2020). Bottlebrush Bridge between Soft Gels and Firm Tissues. ACS Cent. Sci..

[117] Vashahi F., Martinez M.R., Dashtimoghadam E., Fahimipour F., Keith A.N., Bersenev E.A., Ivanov D.A., Zhulina E.B., Popryadukhin P., Matyjaszewski K. (2022). Injectable Bottlebrush Hydrogels with Tissue-Mimetic Mechanical Properties. Sci. Adv..

[118] Wang C.J., Vashahi F., Moutsios I., Umarov A.Z., Ageev G.G., Wang Z., Ivanov D.A., Dobrynin A.V., Sheiko S.S. (2024). Bottlebrush Hydrogels with Hidden Length: Super-Swelling and Mechanically Robust. Adv. Funct. Mater..

[119] Sousa J.P.M., Stratakis E., Mano J., Marques P.A.A.P. (2023). Anisotropic 3D Scaffolds for Spinal Cord Guided Repair: Current Concepts. Biomater. Adv..

[120] Wang Y., Yao M., Zhou J., Zheng W., Zhou C., Dong D., Liu Y., Teng Z., Jiang Y., Wei G. (2011). The Promotion of Neural Progenitor Cells Proliferation by Aligned and Randomly Oriented Collagen Nanofibers through B1 Integrin/MAPK Signaling Pathway. Biomaterials.

[121] Wang H.B., Mullins M.E., Cregg J.M., McCarthy C.W., Gilbert R.J. (2010). Varying the Diameter of Aligned Electrospun Fibers Alters Neurite Outgrowth and Schwann Cell Migration. Acta Biomater..

[122] Xie J., Liu W., MacEwan M.R., Bridgman P.C., Xia Y. (2014). Neurite Outgrowth on Electrospun Nanofibers with Uniaxial Alignment: The Effects of Fiber Density, Surface Coating, and Supporting Substrate. ACS Nano.

[123] Shi M., Xu Q., Ding L., Xia Y., Zhang C., Lai H., Liu C., Deng D.Y.B. (2022). Cell Infiltrative Inner Connected Porous Hydrogel Improves Neural Stem Cell Migration and Differentiation for Functional Repair of Spinal Cord Injury. ACS Biomater. Sci. Eng..

[124] Leong M.F., Chian K.S., Mhaisalkar P.S., Ong W.F., Ratner B.D. (2009). Effect of Electrospun Poly(D,L-Lactide) Fibrous Scaffold with Nanoporous Surface on Attachment of Porcine Esophageal Epithelial Cells and Protein Adsorption. J. Biomed. Mater. Res..

[125] Xuan H., Li B., Xiong F., Wu S., Zhang Z., Yang Y., Yuan H. (2021). Tailoring Nano-Porous Surface of Aligned Electrospun Poly(L-Lactic Acid) Fibers for Nerve Tissue Engineering. IJMS.

[126] Hao W., Han J., Chu Y., Huang L., Sun J., Zhuang Y., Li X., Ma H., Chen Y., Dai J. (2018). Lower Fluidity of Supported Lipid Bilayers Promotes Neuronal Differentiation of Neural Stem Cells by Enhancing Focal Adhesion Formation. Biomaterials.

[127] Silva G.A., Czeisler C., Niece K.L., Beniash E., Harrington D.A., Kessler J.A., Stupp S.I. (2004). Selective Differentiation of Neural Progenitor Cells by High-Epitope Density Nanofibers. Science.

[128] Golabchi A., Wu B., Cao B., Bettinger C.J., Cui X.T. (2019). Zwitterionic Polymer/Polydopamine Coating Reduce Acute Inflammatory Tissue Responses to Neural Implants. Biomaterials.

[129] Yang Q., Wu B., Eles J.R., Vazquez A.L., Kozai T.D.Y., Cui X.T. (2020). Zwitterionic Polymer Coating Suppresses Microglial Encapsulation to Neural Implants In Vitro and In Vivo. Adv. Biosys..

[130] Wang S., Guan S., Sun C., Liu H., Liu T., Ma X. (2023). Electrical Stimulation Enhances the Neuronal Differentiation of Neural Stem Cells in Three-Dimensional Conductive Scaffolds through the Voltage-Gated Calcium Ion Channel. Brain Res..

[131] Lopez-Buenafe G.D.R., Alonso-Cabrera J.A., Marcuello C., Ortiz-Perez M., Benito-Lopez F., Colom A., Basabe-Desmonts L., Saez J. (2025). Fabrication and Characterization of PEDOT:PSS-Based Microstructured Electrodes for In Vitro Cell Culture. Adv. Mater. Inter..

[132] Poulkouras R., Dijk G., Lefevre M., Bača M., Moreau D., O’Connor R.P. (2022). PEDOT:PSS Coated Electrodes Reduce Intracellular Oxidation and Cell Damage with Pulsed Electric Field Application. Bioelectrochemistry.

[133] Yao S., Yang Y., Li C., Yang K., Song X., Li C., Cao Z., Zhao H., Yu X., Wang X. (2024). Axon-like Aligned Conductive CNT/GelMA Hydrogel Fibers Combined with Electrical Stimulation for Spinal Cord Injury Recovery. Bioact. Mater..

[134] Chen P., Xu C., Wu P., Liu K., Chen F., Chen Y., Dai H., Luo Z. (2022). Wirelessly Powered Electrical-Stimulation Based on Biodegradable 3D Piezoelectric Scaffolds Promotes the Spinal Cord Injury Repair. ACS Nano.

[135] Liu M., Zhang W., Han S., Zhang D., Zhou X., Guo X., Chen H., Wang H., Jin L., Feng S. (2024). Multifunctional Conductive and Electrogenic Hydrogel Repaired Spinal Cord Injury via Immunoregulation and Enhancement of Neuronal Differentiation. Adv. Mater..

[136] Shahriari D., Koffler J., Lynam D.A., Tuszynski M.H., Sakamoto J.S. (2016). Characterizing the Degradation of Alginate Hydrogel for Use in Multilumen Scaffolds for Spinal Cord Repair. J. Biomed. Mater. Res..

[137] Tukmachev D., Forostyak S., Koci Z., Zaviskova K., Vackova I., Vyborny K., Sandvig I., Sandvig A., Medberry C.J., Badylak S.F. (2016). Injectable Extracellular Matrix Hydrogels as Scaffolds for Spinal Cord Injury Repair. Tissue Eng. Part A.

[138] Fu K., Pack D.W., Klibanov A.M., Langer R. (2000). Visual Evidence of Acidic Environment Within Degrading Poly(Lactic-*Co*-Glycolic Acid) (PLGA) Microspheres. Pharm. Res..

[139] Mayer J.B., Patil S.M., Shin S.-H., Yoo J., Won Y.-Y. (2025). Internal Water-Induced Acceleration, Chemical Pathways, and Contributing Factors in the Degradation of Poly(Lactic-*Co*-Glycolic Acid) (PLGA) Microparticles and Devices. ACS Biomater. Sci. Eng..

[140] Agarwal M., Koelling K.W., Chalmers J.J. (1998). Characterization of the Degradation of Polylactic Acid Polymer in a Solid Substrate Environment. Biotechnol. Prog..

[141] Pellerin L., Pellegri G., Bittar P.G., Charnay Y., Bouras C., Martin J.-L., Stella N., Magistretti P.J. (1998). Evidence Supporting the Existence of an Activity-Dependent Astrocyte-Neuron Lactate Shuttle. Dev. Neurosci..

[142] Mächler P., Wyss M.T., Elsayed M., Stobart J., Gutierrez R., von Faber-Castell A., Kaelin V., Zuend M., San Martín A., Romero-Gómez I. (2016). In Vivo Evidence for a Lactate Gradient from Astrocytes to Neurons. Cell Metab..

[143] Jeon J., Park S.H., Choi J., Han S.M., Kim H.-W., Shim S.R., Hyun J.K. (2024). Association between Neural Stem/Progenitor Cells and Biomaterials in Spinal Cord Injury Therapies: A Systematic Review and Network Meta-Analysis. Acta Biomater..

[144] Park H.H., Kim Y., Jang B.S., Genişcan S., Hwang D.H., Seo Y., Jee S., Seo H.G., Kim H.S., Einisadr A. (2026). Mechanical Environment Afforded by Engineered Hydrogel Critically Regulates Survival of Neural Stem Cells Transplanted in the Injured Spinal Cord via Piezo1-Mediated Mechanotransduction. Adv. Sci..

[145] Tigner T.J., Dampf G., Tucker A., Huang Y., Jagrit V., Clevenger A.J., Mohapatra A., Raghavan S.A., Dulin J.N., Alge D.L. (2024). Clickable Granular Hydrogel Scaffolds for Delivery of Neural Progenitor Cells to Sites of Spinal Cord Injury. Adv. Healthc. Mater..

[146] Zhang J., Li X., Guo L., Gao M., Wang Y., Xiong H., Xu T., Xu R. (2024). 3D Hydrogel Microfibers Promote the Differentiation of Encapsulated Neural Stem Cells and Facilitate Neuron Protection and Axon Regrowth after Complete Transactional Spinal Cord Injury. Biofabrication.

[147] Roh E.J., Kim D.-S., Kim J.H., Lim C.S., Choi H., Kwon S.Y., Park S.-Y., Kim J.Y., Kim H.-M., Hwang D.-Y. (2023). Multimodal Therapy Strategy Based on a Bioactive Hydrogel for Repair of Spinal Cord Injury. Biomaterials.

[148] Thompson R.E., Pardieck J., Smith L., Kenny P., Crawford L., Shoichet M., Sakiyama-Elbert S. (2018). Effect of Hyaluronic Acid Hydrogels Containing Astrocyte-Derived Extracellular Matrix and/or V2a Interneurons on Histologic Outcomes Following Spinal Cord Injury. Biomaterials.

[149] Zheng Y., Zhang Z., Fu Z., Wang Q., Zhang S., Zhang T., Zhu M., Fan S., Shen Y., Xiang J. (2026). Disrupting CSPG-Driven Microglia–Astrocyte Crosstalk Enables Scar-Free Repair in Spinal Cord Injury. Adv. Sci..

[150] Li X., Zhang J., Zhang Y., Guo L., Gao M., Wang Y., Qiu W., Yuan Y., Zhu J., Liu B. (2025). Conjugated Therapy with Coaxially Printed Neural Stem Cell-Laden Microfibers and Umbilical Cord Mesenchymal Stem Cell Derived Exosomes on Complete Transactional Spinal Cord Defects. Mater. Today Bio.

[151] Chen H., Wang W., Yang Y., Zhang B., Li Z., Chen L., Tu Q., Zhang T., Lin D., Yi H. (2025). A Sequential Stimuli-Responsive Hydrogel Promotes Structural and Functional Recovery of Severe Spinal Cord Injury. Biomaterials.

[152] Li Y., Liang Y., He C., Yao R., Jian K., Shi L. (2025). A Dual-Drug Sequential Delivery Hydrogel for Programmatic Microglia/Macrophage Polarization and Function Recovery in Spinal Cord Injury. Mater. Today Bio.

[153] Yao Y., Dong X., Pang Z., Shao J., He Z., Liu K., Hou P., Hu F., Liu W., Huo Y. (2025). A Zinc-Citrate Metal–Organic Framework-Based Adaptable Hydrogen Sulfide Delivery System for Regulating Neuroregeneration Microenvironment in Spinal Cord Injury. ACS Nano.

[154] Liu F., Fu T.-M. (2025). Material and Design Strategies for Chronically-Implantable Neural Probes. NPG Asia Mater..

[155] Luan L., Wei X., Zhao Z., Siegel J.J., Potnis O., Tuppen C.A., Lin S., Kazmi S., Fowler R.A., Holloway S. (2017). Ultraflexible Nanoelectronic Probes Form Reliable, Glial Scar–Free Neural Integration. Sci. Adv..

[156] Choi Y., Jeon W., Kim Y., Kim H., Cho Y., Jang Y., Lee S., Kim D., Mun T.J., Yoo Y. (2026). Photoinitiated CVD Antifouling Coatings Enable Long-Term Stability of Flexible Multifunctional Neural Probes for Chronic Neural Recording. Biomaterials.

[157] Golabchi A., Woeppel K.M., Li X., Lagenaur C.F., Cui X.T. (2020). Neuroadhesive Protein Coating Improves the Chronic Performance of Neuroelectronics in Mouse Brain. Biosens. Bioelectron..

[158] Minev I.R. (2023). Electronic Tissue Technologies for Seamless Biointerfaces. J. Polym. Sci..

[159] Da Silva A.C., Akbar T.F., Paterson T.E., Werner C., Tondera C., Minev I.R. (2022). Electrically Controlled Click-Chemistry for Assembly of Bioactive Hydrogels on Diverse Micro- and Flexible Electrodes. Macromol. Rapid Commun..

[160] Akbar T.F., Jimenez-Rodriguez C.A., Biktimirova R., Hermes I., Kurth T., Pham M.D., Tsurkan M., Friedrichs J., Morgan F.L.C., Kleemann H. (2026). Conductive Hydrogels for Exogenous Sensing and Cell Fate Control. Adv. Mater..

[161] Zhang J., Yang H., Wu J., Zhang D., Wang Y., Zhai J. (2022). Recent Progresses in Novel in Vitro Models of Primary Neurons: A Biomaterial Perspective. Front. Bioeng. Biotechnol..

[162] Pereira M.F., Shyti R., Testa G. (2024). In and out: Benchmarking in Vitro, in Vivo, Ex Vivo, and Xenografting Approaches for an Integrative Brain Disease Modeling Pipeline. Stem Cell Rep..

[163] Etayo-Escanilla M., Campillo N., Ávila-Fernández P., Baena J.M., Chato-Astrain J., Campos F., Sánchez-Porras D., García-García Ó.D., Carriel V. (2024). Comparison of Printable Biomaterials for Use in Neural Tissue Engineering: An In Vitro Characterization and In Vivo Biocompatibility Assessment. Polymers.

[164] Carnicer-Lombarte A., Chen S.-T., Malliaras G.G., Barone D.G. (2021). Foreign Body Reaction to Implanted Biomaterials and Its Impact in Nerve Neuroprosthetics. Front. Bioeng. Biotechnol..

[165] Urbanski M.M., Kingsbury L., Moussouros D., Kassim I., Mehjabeen S., Paknejad N., Melendez-Vasquez C.V. (2016). Myelinating Glia Differentiation Is Regulated by Extracellular Matrix Elasticity. Sci. Rep..

[166] Segel M., Neumann B., Hill M.F.E., Weber I.P., Viscomi C., Zhao C., Young A., Agley C.C., Thompson A.J., Gonzalez G.A. (2019). Niche Stiffness Underlies the Ageing of Central Nervous System Progenitor Cells. Nature.

[167] Bennett M.L., Bennett F.C., Liddelow S.A., Ajami B., Zamanian J.L., Fernhoff N.B., Mulinyawe S.B., Bohlen C.J., Adil A., Tucker A. (2016). New Tools for Studying Microglia in the Mouse and Human CNS. Proc. Natl. Acad. Sci. USA.

[168] Greenhalgh A.D., David S. (2014). Differences in the Phagocytic Response of Microglia and Peripheral Macrophages after Spinal Cord Injury and Its Effects on Cell Death. J. Neurosci..

[169] Shechter R., London A., Varol C., Raposo C., Cusimano M., Yovel G., Rolls A., Mack M., Pluchino S., Martino G. (2009). Infiltrating Blood-Derived Macrophages Are Vital Cells Playing an Anti-Inflammatory Role in Recovery from Spinal Cord Injury in Mice. PLoS Med..

[170] Göritz C., Dias D.O., Tomilin N., Barbacid M., Shupliakov O., Frisén J. (2011). A Pericyte Origin of Spinal Cord Scar Tissue. Science.

[171] Soderblom C., Luo X., Blumenthal E., Bray E., Lyapichev K., Ramos J., Krishnan V., Lai-Hsu C., Park K.K., Tsoulfas P. (2013). Perivascular Fibroblasts Form the Fibrotic Scar after Contusive Spinal Cord Injury. J. Neurosci..

[172] Dias D.O., Kim H., Holl D., Werne Solnestam B., Lundeberg J., Carlén M., Göritz C., Frisén J. (2018). Reducing Pericyte-Derived Scarring Promotes Recovery after Spinal Cord Injury. Cell.

[173] Taylor A.M., Blurton-Jones M., Rhee S.W., Cribbs D.H., Cotman C.W., Jeon N.L. (2005). A Microfluidic Culture Platform for CNS Axonal Injury, Regeneration and Transport. Nat. Methods.

[174] Park J., Koito H., Li J., Han A. (2009). Microfluidic Compartmentalized Co-Culture Platform for CNS Axon Myelination Research. Biomed. Microdevices.

[175] Perea G., Yang A., Boyden E.S., Sur M. (2014). Optogenetic Astrocyte Activation Modulates Response Selectivity of Visual Cortex Neurons in Vivo. Nat. Commun..

[176] Zeisel A., Muñoz-Manchado A.B., Codeluppi S., Lönnerberg P., La Manno G., Juréus A., Marques S., Munguba H., He L., Betsholtz C. (2015). Cell Types in the Mouse Cortex and Hippocampus Revealed by Single-Cell RNA-Seq. Science.

[177] Stuart T., Butler A., Hoffman P., Hafemeister C., Papalexi E., Mauck W.M., Hao Y., Stoeckius M., Smibert P., Satija R. (2019). Comprehensive Integration of Single-Cell Data. Cell.

